# The Role of Ferroptosis in Osteoporosis and Advances in Chinese Herbal Interventions

**DOI:** 10.3390/biology14040367

**Published:** 2025-04-02

**Authors:** Pan Li, Tian-Yang Xu, Ao-Xue Yu, Jing-Ling Liang, Ya-Shuang Zhou, Huai-Zhu Sun, Yu-Lin Dai, Jia Liu, Peng Yu

**Affiliations:** 1 College of Pharmacy, Changchun University of Chinese Medicine, Jilin 130117, China; 22203070604@stu.ccucm.edu.cn (P.L.); 22203089313@stu.ccucm.edu.cn (A.-X.Y.); 21103080001@stu.ccucm.edu.cn (J.-L.L.); 22203089705@stu.ccucm.edu.cn (H.-Z.S.); 2 Innovation Practice Center, Changchun University of Chinese Medicine, Jilin 130117, China; xuty@ccucm.edu.cn; 3 Ginseng Scientific Research Institute, Jilin 130117, China; daiyl@ccucm.edu.cn; 4 Innovation and Entrepreneurship College, Changchun University of Chinese Medicine, Jilin 130117, China

**Keywords:** ferroptosis, osteoporosis, herbal interventions

## Abstract

OP, a systemic bone disorder marked by reduced bone mass and heightened fracture risk, poses a significant global health burden, particularly among aging populations. Current treatments, including bisphosphonates and calcium supplementation, are limited by adverse effects and incomplete efficacy. Emerging research highlights ferroptosis—an iron-dependent cell death driven by lipid peroxidation—as a critical contributor to OP pathogenesis, characterized by dysregulated iron metabolism, oxidative stress, and lipid peroxide accumulation, which disrupt bone remodeling by impairing osteoblast function and enhancing osteoclast activity. This review elucidates the mechanistic interplay between ferroptosis and OP subtypes (diabetic osteoporosis (DOP), glucocorticoid-induced (GIOP), and postmenopausal osteoporosis (PMOP)) and evaluates the efficacy of Chinese herbal interventions in mitigating ferroptosis-driven bone loss. Key findings reveal that excess iron exacerbates lipid peroxidation via the Fenton reaction, while glutathione peroxidase 4 (GPX4) inactivation and system Xc- inhibition amplify oxidative damage. In DIOP, hyperglycemia-induced ROS and advanced glycation end products suppress osteogenesis, countered by melatonin and naringenin via nuclear factor -related factor 2 (Nrf2)/GPX4 activation. GIOP involves dexamethasone-mediated GPX4 downregulation, mitigated by exosomes and melatonin through phosphatidylinositol 3-kinase (PI3K)/protein kinase B (AKT)/mammalian target of rapamycin (mTOR) signaling. PMOP driven by estrogen deficiency-induced iron overload is alleviated by aconitine and icariin (ICA) via nuclear factor kappa-light-chain-enhancer of activated B cells (NF-κB) and signal transducer and activator of transcription 3 (STAT3) pathways. Chinese herbs, including active compounds (quercetin, gastrodin, ICA, etc.) and formulations (Bugu Shengsui Capsule, Erxian Decoction (EXD), etc.), regulate iron metabolism, enhance antioxidant defenses (Nrf2/heme oxygenase 1(HO-1)), and inhibit lipid peroxidation, effectively restoring bone homeostasis. These findings underscore ferroptosis as a pivotal mechanism in OP progression and highlight the therapeutic promise of Chinese herbs in bridging traditional medicine with modern mechanistic insights. Future research should prioritize elucidating precise molecular targets, optimizing formulations, and validating clinical efficacy to address current therapeutic gaps.

## Simple Summary

Ferroptosis stands as a unique modality of cell death, distinctly different from conventional apoptosis, necrosis, and other cellular demise pathways. Primarily induced by the surfeit of intracellular iron and the accumulation of reactive oxygen species (ROS), it has also been termed iron-dependent oxidative cell death. Osteoporosis (OP) is a widespread bone disorder. In recent years, a surge in research has revealed that interventions involving Chinese herbs can regulate ferroptosis, presenting novel strategies for the management of bone ailments, including OP. The aim of this paper is to elucidate the intricate relationship between ferroptosis and OP by delving into the fundamental mechanisms underlying ferroptosis. Additionally, we endeavor to dissect the latest advancements in how Chinese herbs can prevent and treat OP through the modulation of ferroptosis, thereby paving the way for innovative strategies that harness the potential of Chinese herbal medicine in the prevention and treatment of OP.

## 1. Introduction

OP, a debilitating systemic bone disease, has garnered growing attention within the medical community. Often termed a “silent epidemic”, it progresses insidiously, with patients typically remaining unaware of OP until they manifest. As the global population ages, its incidence is rising steadily, making it a significant health concern impacting the quality of life of the middle-aged and elderly [1,2,3]. It is estimated that OP affects approximately 33% of women and 20% of men aged 50 and older globally [4]. In the management of OP, calcium and vitamin D supplementation form the cornerstone of treatment [5]. Pharmacotherapy is equally essential for enhancing bone mass and diminishing fracture risk. Among the therapeutic agents, bisphosphonates are predominantly used in clinical practice [6]. However, their adverse effects and impact on patient compliance are critical considerations. For instance, alendronate may irritate the digestive tract [7], while zoledronic acid and teriparatide are associated with adverse reactions such as nausea, vomiting, flushing, and localized inflammation [8]. Moreover, osteoporotic fractures impose a substantial economic burden on society and families due to their high incidence, mortality, and the exorbitant medical costs involved, in addition to severely compromising patients’ quality of life. OP is characterized by reduced bone mass, deterioration of the bone tissue’s microarchitecture, heightened bone fragility, and an elevated risk of fractures. Addressing this condition necessitates advancing our understanding and developing efficacious therapeutic strategies to counteract its detrimental effects [9,10]. The pathological hallmarks of OP encompass several critical aspects [11]: (1) Reduction in Bone Mass: This includes a decrease in the proportion of bone minerals and extra-cellular matrices, contributing to the overall weakening of the bone structure. (2) Imbalance in Bone Tissue Resorption and Formation: This imbalance leads to the degradation of the bone microstructure, manifesting as the disruption, deformation, and fracturing of the trabecular bone architecture. (3) Increased Bone Fragility and Decreased Bone Strength: This results in a higher incidence of fractures, both in the form of microfractures and complete fractures, and a reduced capacity to bear load, further exacerbating the condition. Current treatments for OP can only delay its onset, reduce bone loss, and lower fracture risk; none can cure the disease [12,13]. Given these limitations, further research into OP’ molecular mechanisms and development is crucial. This will facilitate the discovery of novel therapeutic approaches and the refinement of substitutes.

Iron plays a pivotal role in the synthesis of numerous essential proteases and is in-dispensable for the body’s vital activities [14,15]. It is intricately involved in a wide array of biochemical processes, encompassing oxygen transport, enzymatic catalysis, and immune responses, and it plays a vital role in energy metabolism disorders, inflammation, oxidative stress, and the maintenance of the homeostatic balance of the body’s internal environment [16,17]. As research advances, it has become increasingly apparent that a spectrum of physiological and pathological processes—including tumor progression, Parkinson’s disease, atherosclerosis, viral infections, OP, immune reactions, and ischemia-reperfusion injury—are intimately linked to ferroptosis. This emerging insight underscores the profound significance of iron in these conditions and the potential ramifications for disease treatment and management strategies [18,19,20,21]. Ferroptosis is emerging as a focal point of research in the treatment of various diseases. Iron metabolism is crucial to bone homeostasis [22,23]. Iron excess can contribute to OP [24], as osteoclasts need more iron for mitochondrial function to inhibit bone resorption. Meanwhile, iron excess [25] can inhibit osteoblasts.

Therapeutic strategies targeting ferroptosis regulation for OP treatment have garnered significant attention. Numerous non-herbal therapeutic agents have demonstrated remarkable efficacy in treating OP through ferroptosis modulation [26,27], including iron chelators such as deferoxamine (DFO) [24,28], deferiprone (DFP) [29], and deferasirox (DFS) [30], as well as MT [31], ferrostatin-1 [32], and vitamin D [33]. While these non-herbal therapeutic agents approaches are characterized by single-target mechanisms and rapid onset of action, conventional clinical anti-OP medications still face limitations such as unstable efficacy, severe adverse effects, and drug resistance [34]. Recent advancements in natural product research across East Asian countries have revealed that, compared to synthetic drugs, natural Chinese herbs exhibit distinct advantages including higher molecular weights, stable active scaffolds, and superior bioactivity in anti-OP processes [35,36,37].

In recent years, the regulatory mechanisms underpinning ferroptosis in the context of OP have garnered escalating attention. Research has elucidated that the ferroptosis process in OP is intricately intertwined with the interplay of diverse mechanisms, including iron ion homeostasis, oxidative stress responses, and lipid peroxide accumulation. A profound comprehension of these mechanisms, along with the inhibition of ferroptosis, holds the key to advancing potent therapeutic drugs and methodologies for the prevention and treatment of OP. This article delves into the mechanisms of ferroptosis, exploring its intricate connection with OP and highlighting groundbreaking research into the role of Chinese herbs in mitigating OP through the modulation of ferroptosis. These insights have the potential to pave the way for innovative approaches and methodologies in the treatment of OP, offering promising avenues for future research and clinical interventions.

## 2. The Mechanism of Ferroptosis

**Figure 1:**
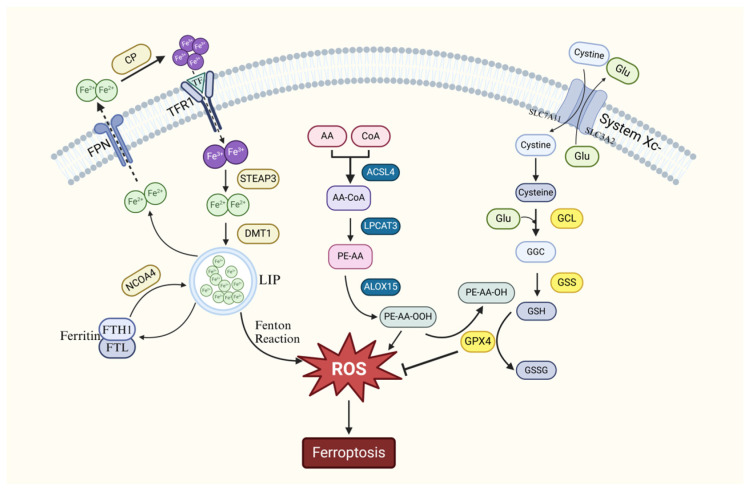
Mechanism of ferroptosis signaling pathway. As shown in the diagram, there are three ways (from left to right): 1. Iron Metabolism Pathway: Dysregulated iron homeostasis (e.g., iron overload or aberrant iron metabolism regulatory proteins) exacerbates lipid peroxidation via the Fenton reaction, which catalyzes reactive oxygen species (ROS) generation. 2. Lipid Peroxide Accumulation Pathway: Glutathione peroxidase 4 (GPX4) maintains redox balance by reducing lipid peroxides. Its inactivation leads to lethal lipid peroxide accumulation, triggering ferroptosis. 3. Cellular Oxidative Stress Pathway: System Xc- mediates cystine uptake for glutathione biosynthesis. Blockade of this transporter results in glutathione depletion, subsequent GPX4 inactivation, and uncontrolled lipid peroxidation, ultimately inducing ferroptosis.

Ferroptosis represents a novel and distinct form of iron-dependent cell death, differing from apoptosis, necrosis, and autophagy. It is primarily characterized by the accumulation of intracellular iron ions and lipid peroxides, playing a pivotal role in both the pathogenesis and therapeutic strategies of a wide range of diseases. The underlying mechanisms of ferroptosis are predominantly centered on abnormal iron metabolism, cellular oxidative stress responses, and the peroxidation of lipids. The mechanism of ferroptosis is shown in Figure 1.

### 2.1. Ferroptosis Caused by Abnormal Iron Metabolism

Iron, an essential trace element for human physiology, has been identified as a standalone risk factor for OP, with disruptions in iron metabolism raising the risk of various skeletal diseases, particularly OP. Primarily existing within the body as ferrous ions (Fe^2+^) and ferric ions (Fe^3+^), the maintenance of intracellular iron homeostasis is contingent upon the balanced processes of iron absorption, export, utilization, and storage. Key proteins involved in iron import include transferrin (Tf), transferrin receptor 1 (TFR1), and divalent metal ion transporter-1 (DMT-1), while iron export is primarily facilitated by membrane iron transport proteins (FPNs). The liberated Fe^2+^ ions are then either sequestered by ferritin (FER) for intracellular storage or expelled by FPNs. FER is composed of two subunits: the ferritin heavy chain 1 (FTH1), which possesses ferrous oxidase activity and is responsible for converting Fe^2+^ to Fe^3+^ for secure storage, and the ferritin light chain (FTL), which does not possess this enzymatic activity.

Circulating Fe^3+^ bind to Tf and are transported into the cell after being recognized by the TFR1 on the cell membrane [38]. Within the cell, the Fe^3+^ is reduced to Fe^2+^ by prostate six-membrane epithelial antigen 3 (STEAP3), which facilitates the release of Fe^2+^ into the intracellular pool of labile iron (LIP) via DMT-1 [39]. Iron participates in energy metabolism, enzymatic reactions, and various other cellular functions. Surplus iron is either expelled from the cell or stored within ferritin [40]. Under normal conditions, the body meticulously regulates iron metabolism, maintaining a dynamic equilibrium between iron intake and output to preserve physiological functions; however, an excess of iron can lead to tissue damage and overall bodily harm [41]. Intracellularly, iron predominantly exists as Fe^2+^ within LIP. The instability and high reactivity of Fe^2+^ enable it to generate hydroxyl radicals through the Fenton reaction, which can react directly with polyunsaturated fatty acids in cellular and plasma membranes, producing a substantial amount of ROS, ultimately leading to cell death. During ferroptosis, Tf carries Fe^3+^, binds to TFR on the plasma membrane, and forms vesicles that transport Fe^3+^ from circulating transferrin into the cell; the low pH within these vesicles prompts the release of Fe^3+^ from Tf. The released Fe^3+^ is then reduced to Fe^2+^ in the cytoplasm, either entering the LIP pool or binding to ferritin [10]. Eventually, ferritin can be engulfed by autophagic lysosomes mediated by nuclear receptor coactivator 4 (NCOA4) [42,43]. Ferritin sequesters intracellular iron in an inert form, which does not contribute to lipid peroxidation [44]. Consequently, the quantity of ferritin determines the cell’s vulnerability to ferroptosis. When LIP is depleted, a higher presence of ferritin means more iron is stored, enhancing resistance to ferroptosis. On the flip side, ferritin depletion releases iron into LIP, increasing sensitivity to ferroptosis [45]. Moreover, a recent study has discovered that prominin 2 aids in combating ferroptosis by promoting the formation of multivesicular bodies and exosomes containing iron-rich proteins that export iron from the cell [46].

### 2.2. Ferroptosis Caused by Oxidative Stress in Cells

Mitochondria, serving as the principal regulators of oxidative phosphorylation, hold significant importance in the realm of oxidative stress and are key generators of ROS. Iron penetrates the mitochondrial matrix by traversing both the outer and inner mitochondrial membranes, thereby modulating the physiological functions of these crucial organelles. Within the context of ferroptosis, mitochondria play an essential role in its regulation. Specifically, erastin triggers the opening of voltage-dependent anion channel proteins 2/3 (VDAC2/3), which are situated on the outer mitochondrial membrane, leading to iron accumulation within the mitochondria. Nevertheless, the precise mechanisms underlying the actions of erastin and VDAC2/3 remain subjects of ongoing investigation [47,48,49].

GPX4, a pivotal and distinctive biomarker of ferroptosis, catalyzes the breakdown of lipid peroxides into fatty alcohols and the conversion of hydrogen peroxide into water. Employing the small molecule inhibitor (1S,3R)-RSL3 (RSL3) to target GPX4, as well as the genetic knockout of GPX4, can effectively trigger ferroptosis [50]. L-glutathione (L-GSH), an antioxidant crucial in managing oxidative stress, exists within cells as reduced glutathione (GSH) and oxidized glutathione (GSSG), comprising glycine, glutamic acid, and cysteine. The GPX4-mediated degradation of lipid peroxides is dependent on GSH to supply electrons throughout the process [51], and the uptake of cystine, essential for GSH synthesis, is facilitated by the sodium-dependent system Xc- (system Xc-), a disulfide-linked heterodimer consisting of a heavy chain (4F2hc, gene name Solute Carrier Family 3 Member 2 (SLC3A2)) and a light chain (xCT, gene name Recombinant Solute Carrier Family 7, Member 11 (SLC7A11)) [52]. Chen et al. [53] clarified the molecular underpinnings of oxidative stress-induced ferroptosis by uncovering the signaling pathways that initiate lipid peroxidation and iron overload. They also identified mitochondrial iron overload, driven by the translocation of HO-1 to the mitochondria, as a contributing mechanism to oxidative stress-induced ferroptosis. Their findings suggest that addressing mitochondrial iron overload or lipid ROS accumulation could offer a novel protective strategy against oxidative stress-induced ferroptosis. Another study [54] demonstrated that a high-iron diet elevates liver iron levels, depletes GSH, and enhances lipid peroxidation and oxidative stress. Moreover, excessive dietary iron reduces the mRNA and protein levels of GPX4 and the cystine-glutamic acid reverse transporter SLC7A11, while increasing the mRNA and protein levels of acyl-CoA synthetase long-chain family member 4 (ACSL4), all of which are indicative of ferroptosis. Ferroptosis further exacerbates lipid peroxidation and mitochondrial ROS production and diminishes matrix metalloproteinase (MMP). This conclusion establishes a direct link between mitochondrial oxidative stress and ferroptosis, potentially through the Nrf2-antioxidant response element (ARE) pathway.

### 2.3. Ferroptosis Caused by Abnormal Lipid Peroxide Metabolism

Lipid peroxidation plays a significant role in ferroptosis. Recent studies have demonstrated that lipid peroxides can undermine the stability of lipid bilayers, ultimately leading to the disintegration of cell membranes. Lipid omics analyses have revealed that ferroptosis-associated lipids, such as arachidonic acid (AA) and Adrenaline (Ad) containing phosphatidylethanolamine, are prone to spontaneous peroxidation in the presence of hydroxyl radicals. These radicals are generated through the Fenton reaction involving redox-active divalent iron and hydrogen peroxide. The susceptibility of polyunsaturated fatty acids (PUFAs) to lipid peroxidation is due to the highly reactive hydrogen atoms present in their methylene bridges [55]. PUFAs in membrane phospholipids can directly engage with hydroxyl radicals, leading to the formation of lipid peroxides that attack cell membranes and induce the morphological changes characteristic of ferroptosis. The decomposition products of lipid peroxides, including malondialdehyde (MDA) and 4-Hydroxynonenal (4-HNE), can interact with nucleic acids and proteins, exacerbating cellular damage. These compounds also serve as effective molecular markers for the detection of ferroptosis and lipid peroxidation [56,57]. The exogenous administration of oleic acid (OA), a monounsaturated fatty acid (MUFA), effectively inhibits erastin-induced ferroptosis by competing with PUFAs for incorporation into phospholipids (PL) [58]. Another study [59] has shown that downregulating the expression of ACSL4 and lysophosphatidylcholine acyltransferase 3 (LPCAT3) can mitigate the accumulation of intracellular lipid peroxide substrates, thereby inhibiting ferroptosis. Furthermore, recent studies have indicated that p53 can suppress the uptake of cystine by system Xc- by downregulating the SLC7A11 subunit. This reduction in cystine-dependent glutathione peroxidase activity and cellular antioxidant capacity leads to an increase in lipid ROS, culminating in cellular ferroptosis [60,61,62,63].

### 2.4. Relationship Between Ferroptosis and Autophagy

Although preliminary studies have shown that ferroptosis is different from autophagy and other types of cell death, recent studies have shown that the activation of ferroptosis depends on the induction of autophagy [64,65]. The mechanism of autophagy promoting ferroptosis involves multiple levels of regulation. The core is to enhance lipid peroxidation and iron ion accumulation by selectively degrading key regulatory proteins or regulating iron metabolism, and ultimately trigger iron-dependent cell death [66,67]. Autophagy releases free iron stored in ferritin by degrading ferritin. Free iron produces ROS through the Fenton reaction, which directly attacks polyunsaturated fatty acids in the cell membrane, triggering a lipid peroxidation chain reaction, leading to cell membrane disintegration and ferroptosis [43,44,68]. This process is mediated by NCOA4. As a selective autophagy receptor for ferritin, NCOA4 promotes the formation of ferritin-autophagosome complex, thereby regulating intracellular iron levels.

## 3. Relationship Between Ferroptosis and Osteoporosis

**Figure 2:**
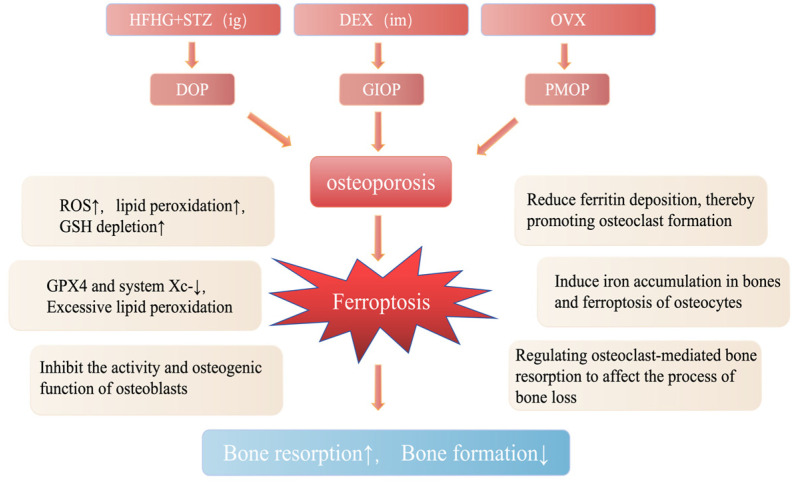
The characteristics of ferroptosis in osteoporosis. Osteoporosis associated with ferroptosis primarily encompasses three subtypes: Diabetic Osteoporosis (DOP), Glucocorticoid-Induced Osteoporosis (GIOP), and Postmenopausal Osteoporosis (PMOP). DOP is typically modeled in preclinical studies using a high-glucose/high-fat (HGHF) diet combined with streptozotocin (STZ) injections to mimic hyperglycemia and metabolic dysregulation. GIOP is simulated via intramuscular dexamethasone (DEX) administration to replicate glucocorticoid excess. PMOP is commonly modeled through bilateral ovariectomy (OVX) in rodents to induce estrogen deficiency. During these processes, ferroptosis disrupts bone homeostasis via the iron metabolism-oxidative stress-lipid peroxidation axis, leading to reduced osteogenesis and enhanced osteoclast-mediated bone resorption. Mechanistically, iron overload and redox imbalance drive lipid peroxidation, impairing osteoblast survival/function while promoting osteoclast activation, ultimately accelerating bone loss.

OP can be broadly categorized into two primary types based on its underlying etiology: primary and secondary OP. Primary OP encompasses a spectrum of conditions, including PMOP, senile OP, and idiopathic OP. Conversely, secondary OP arises due to various identifiable factors, such as bone metabolic diseases, medications, or other specific causes, including GIOP and DOP. Notably, significant associations have been observed between ferroptosis and several types of OP, notably DOP, GIOP, and PMOP. The relationship between ferroptosis and OP is shown in Figure 2.

### 3.1. Diabetic Osteoporosis

The global health landscape is significantly challenged by the prevalence of diabetes and its associated complications. It is estimated that approximately one in every eleven adults aged between 20 and 79 worldwide is affected by diabetes, with type 2 diabetes accounting for 90% of these cases [69]. The link between diabetes and OP is well-established, with the latter being influenced by a multitude of factors [70]. Research indicates that reduced bone formation, a key aspect of OP, can be attributed to oxidative stress induced by elevated glucose levels and the accumulation of advanced glycation end products (AGEs) within collagen [71]. Additionally, studies have demonstrated that diminished levels of insulin and insulin-like growth factor 1 (IGF-1) can impair osteogenic activity, thereby contributing to the development of OP [72]. Furthermore, certain antidiabetic medications, such as thiazolidinediones, have been implicated in adversely affecting bone metabolism and increasing the risk of fractures [73,74]. Diabetic patients frequently experience heightened oxidative stress, a condition linked to elevated blood glucose levels and the buildup of AGEs. This oxidative stress can precipitate ferroptosis, as it encompasses the generation of ROS that assail polyunsaturated fatty acids within the cell membrane. This assault results in lipid peroxidation and ultimately cell death [75,76]. Ferroptosis is marked by the accumulation of intracellular iron ions and lipid peroxides. In the context of DOP, disruptions in iron metabolism may precipitate the accumulation of intracellular iron ions, thereby initiating ferroptosis and impacting the survival and functionality of bone cells [77].

Both in vivo and in vitro experiments [78] have clearly discovered that high-glucose (HG) conditions can trigger ferroptosis in DOP through the enhancement of ROS, lipid peroxidation, and glutathione depletion. Conversely, melatonin has been shown to significantly reduce ferritin deposition and enhance the osteogenic potential of mouse embryonic pre-osteoblasts (MC3T3-E1) cells by activating the Nrf2/HO-1 signaling pathway. And another study [65] showed that HG could induce ferroptosis in a concentration-dependent manner to inhibit the viability and osteogenic function of osteoblasts. A recent study [79] in 2022 revealed that disrupting the self-sustaining cycle of lipid peroxidation and HO-1 activation can effectively reduce ferroptosis in osteocytes during DOP, leading to an improvement in trabecular bone deterioration. Additionally, another investigation [80] demonstrated that MaR1 therapy, a novel pro-resolving mediator derived from docosahexaenoic acid, can regulate the Nrf2 pathway through modulation of SLC7A11/GPX4 signaling. This, in turn, protects osteoblasts from ferroptosis and enhances the osteogenic process in DOP. Researchers [81] established a mouse model of DOP by administering streptozotocin (STZ) injections combined with a HG, high-fat (HF) diet. and cultured bone marrow mesenchymal stem cells (BMSCs) under HG conditions to replicate the diabetic milieu in vitro. Their findings demonstrated that vitamin K2 (VK2) can alleviate DOP by activating the Adenosine 5‘-monophosphate (AMP)-activated protein kinase (AMPK)/Silent mating type information regulation 2 homolog-1 (SIRT1) signaling pathway, thereby inhibiting ferroptosis. Similarly, XU and colleagues [82] have demonstrated that Polydatin (Pol, the main effective components from Chinese herba *Polygonum cuspidatum* Sieb. et Zucc.) exhibits a marked efficacy in ameliorating HGHF-induced bone degradation and ferroptosis. This effect is manifested through an elevation in GSH levels, a reduction in MDA levels, lipid peroxidation, and mitochondrial ROS. Furthermore, Pol was found to enhance bone mineral density and GPX4 labeling while decreasing ROS levels in the distal femur. These findings provide compelling evidence that Pol can inhibit ferroptosis by activating the Nrf2/GPX4 signaling pathway, thereby effectively preventing DOP.

### 3.2. Glucocorticoid-Induced Osteoporosis

The nexus between GIOP and ferroptosis is garnering escalating interest. Glucocorticoids, a group of steroid hormones, are extensively utilized in the management of autoimmune and inflammatory diseases [83,84]. Nonetheless, since their approval for clinical trials in 1955 [85], a growing body of evidence has demonstrated that the prolonged or high-dose administration of glucocorticoids can precipitate a spectrum of adverse effects, notably OP [86]. Statistics reveal that over 10% of patients undergoing long-term glucocorticoid therapy have been diagnosed with OP [87]. GIOP is recognized as the predominant form of secondary OP [88]. It exacerbates the progression of OP by impacting the differentiation and functionality of osteoblasts, enhancing bone resorption, and diminishing bone formation [89]. Moreover, the connection between GIOP and ferroptosis has been progressively uncovered. Ferroptosis is marked by the accumulation of intracellular iron ions and lipid peroxides. In the context of GIOP, disruptions in iron metabolism may result in the accumulation of intracellular iron ions, thereby initiating ferroptosis and impacting the viability and functionality of bone cells. Recent research has also indicated that glucocorticoids can induce oxidative stress in osteoblasts and escalate the generation of ROS, which is intricately associated with the induction of ferroptosis. Furthermore, glucocorticoids are known to augment the incidence of ferroptosis by suppressing the activity of antioxidant enzymes, including GPX4.

GIOP is intimately linked to ferroptosis, a form of cell death characterized by excessive lipid peroxidation due to the downregulation of GPX4 and the system Xc-. Dexamethasone (DEX), a commonly utilized glucocorticoid for treating inflammatory and autoimmune diseases [43,90] has been instrumental in studying this relationship. Studies have [91] employed a high-dose DEX regimen to establish a GIOP model in mice, revealing that high-dose DEX downregulated GPX4 and system Xc-, escalated lipid peroxidation levels, and induced metabolic disruptions concerning glutamate and cysteine. These findings suggest that DEX therapy can precipitate ferroptosis in osteoblasts. Prolonged and high-dose use of steroid hormones has been found to reduce antioxidant capacity, impair osteoblast activity and function, and contribute to the development of OP and osteonecrosis. The application of exosomes derived from endothelial cells (EC-Exos) has been shown to inhibit DEX-induced osteoblast apoptosis and ferroptosis, thereby slowing the progression of OP [69]. Additionally, research has identified that DEX triggers ferroptosis in MC3T3-E1 cells through the protein 53 (p53)/SLC7A11/GPX4 pathway, providing new insights into the molecular mechanisms underlying glucocorticoid-induced osteonecrosis [92]. Meanwhile, studies in zebrafish models have demonstrated that DEX administration leads to OP in 3dfp zebrafish larvae and hinders caudal fin regeneration, establishing a connection between ferroptosis and DEX-induced toxicity [93]. Furthermore, research has [94] confirmed the close relationship between glucocorticoid-induced ferroptosis and GIOP, and showed that melatonin (MT) can inhibit ferroptosis by activating the PI3K/AKT/mTOR signaling pathway, thus preventing the onset of GIOP.

### 3.3. Postmenopausal Osteoporosis

PMOP arises from estrogen deficiency in women who have undergone menopause. This deficiency is linked to inadequate osteoblast differentiation and heightened osteoclast activity, culminating in diminished bone mass and heightened bone fragility. Studies have demonstrated that serum ferritin levels are markedly increased in postmenopausal women, a condition that is closely correlated with reduced bone mineral density [95,96] Ferroptosis, a form of necrosis governed by iron-dependent cell death mechanisms, is influenced by estrogen withdrawal, which can disrupt iron metabolism—a factor implicated in the pathogenesis of PMOP [97].

An in vivo pharmacological study [98] developed a PMOP model using bilaterally ovariectomized (OVX) mice, observing that the expression of interferon regulatory factor 9 (IRF9) was diminished in these mice, which exhibited hyperactive osteoclasts. In vitro IRF9 knockout experiments revealed that the absence of IRF9 could enhance osteoclast differentiation by decreasing ferritin deposition, with further evidence suggesting that this reduction in ferritin was mediated by the activation of STAT3, consequently fostering osteoclast formation. Another study [99] discovered that aconitine (The main effective components from Chinese herba *Aconitum carmichaelii* Debeaux) could modulate the expression of GPX4 and ACSL4 by suppressing the NF-κB signaling pathway in OVX model mice, thereby inhibiting ferroptosis in osteoclasts. Furthermore, research [100] has demonstrated that estrogen deficiency could lead to iron accumulation in bone tissue and ferroptosis in osteocytes, which in turn resulted in reduced bone mineral density. Their study identified ferroptosis in osteocytes from patients with PMOP, indicating that ferroptosis in these cells influences bone loss through the regulation of osteoclast-mediated bone resorption, with Nrf2 being a pivotal regulatory pathway in ferroptosis-induced bone loss in osteocytes.

## 4. Chinese Herbs Prevents Osteoporosis by Intervening Ferroptosis

Traditional Chinese medicine (TCM), a time-honored and respected medical resource, offers unique therapeutic advantages and holds vast potential for diverse applications. A multitude of Chinese herbs demonstrate a wide array of effects, encompassing anti-oxidative, anti-inflammatory, and anti-apoptotic properties. These herbs possess the remarkable capability to interfere with the ferroptosis process by subtly and precisely regulating both internal and external cellular environmental factors. Here, we enumerate the role of Chinse Herbs and Chinese Herbal Remedies in intervening in OP through iron death, of which, Chinse Herbs are shown in Table 1 and Chinese Herbal Remedies are shown in Table 2.

### 4.1. Chinese Herbs

#### 4.1.1. Eucommiae Cortex

Eucommiae cortex, derived from the dried bark of the *Eucommia ulmoides* Oliv. of Eucommiaceae plant, which was first recorded and documented in “Shennong Bencao Jing”, a classical text on TCM [119]. Previous literature mentioned that its virtues in alleviating waist and spine pain, serving as a tonic, replenishing essence, and fortifying bones and muscles [120,121,122]. These descriptions suggest that Eucommiae cortex has long been employed in the treatment of OP-related symptoms, including low back pain and skeletal issues. The 2020 edition of the “Chinese Pharmacopoeia” further attests to its medicinal properties, noting its efficacy in nourishing the liver and kidney, fortifying bones and muscles, as well as safeguarding against miscarriages [123]. This herbal remedy is frequently prescribed for addressing conditions such as liver and kidney deficiency, lumbar and knee discomfort, muscular weakness, dizziness, pregnancy-related bleeding, and fetal instability, which can effectively prevent bone loss, improve bone biomechanical strength, prevent the deterioration of trabecular microstructure and cure OP [124,125,126,127].

Prior research has validated the presence of quercetin and Eucommiae cortex polysaccharide as its active constituents, which exhibit efficacy in preventing OP through modulation of ferroptosis. Quercetin (QUE) has a wide range of sources and exists in a variety of TCM and foods, such as Radix Scutellariae, Flos Lonicerae, Radix Puerariae, Flos Chrysanthemi, Onion, and Apple, etc. One research [101] discovered that QUE (2.5 μM and 5 μM) can enhance alkaline phosphatase (ALP) activity in MC3T3-E1 cells during ferroptosis, promotes the formation of bone mineralization nodules, and upregulates the expression of the Runt-related transcription factor 2 (Runx2) and Osterix (Osx). Furthermore, QUE alleviates FAC-induced apoptosis and ROS production by downregulating the expression of cysteinyl aspartate specific proteinase 3 (Caspase3) and BCL-2-associated X protein (Bax), while upregulating B-cell lymphoma-2 (Bcl-2). The results of the in vivo study showed that QUE (50 mg/kg/d and 100 mg/kg/d) was able to reduce iron deposition induced by iron dextrose and attenuate bone loss. This mechanism helps prevent ferroptosis-induced OP through the activation of the Nrf2/HO-1 signaling pathway. An in vivo experimental study [102] determined that polysaccharides derived from Eucommia cortex (100 mg/kg/d and 300 mg/kg/d) enhance ERK phosphorylation, inhibit Jun N-terminal kinase (JNK) phosphorylation, activate Nrf2, elevate the expression of NAD(P)H quinone dehydrogenase 1 (NQO-1), counteract oxidative stress, and enhance osteogenic function through the ERK/JNK/Nrf2 pathway. Moreover, Eucommia cortex polysaccharide (100 mg/kg/d and 300 mg/kg/d) promotes osteoblast differentiation to enhance bone formation, thus improving bone microarchitecture destruction caused by glucocorticoids, which are regulated by ERK/bone morphogenetic protein 2 (BMP2)/Smad proteins (SMAD) signaling [103]. Another study showed that 10 μM Aucubin (AU) stimulated the proliferation of BMSCs and enhanced osteogenic differentiation of BMSCs by protecting against ferroptosis and promoting the activation of BMP2/SMAD signaling. Meanwhile, animal experiments conducted in vivo have demonstrated the ability of injecting 30 mg/kg/d AU to enhance bone regeneration in OVX rats and mitigate the advancement of OP [104].

#### 4.1.2. Gastrodia Rhizoma

Gastrodia rhizoma is derived from the dried tuber of *Gastrodia elata* Bl. of orchid plant, which was mentioned in the “Compendium of Materia Medica” as indications of Gastrodia rhizoma: migraine, stroke, hand and foot, muscle and bone pain, walking difficulties, heavy waist and knees, etc. These records show that Gastrodia rhizoma has the effects of replenishing qi and strengthening muscles and bones, which may have a certain relieving effect on the symptoms of bone pain and heavy waist and knees in patients with OP. The 2020 edition of the “Chinese Pharmacopoeia” points out that it can effectively alleviates wind and spasms, calms liver yang, dispels wind, and promotes the unobstructed flow of qi and blood through the meridians. In clinical practice, it is commonly prescribed to treat conditions such as infantile convulsions, epileptic seizures, tetanus, headaches, dizziness, weakness in the hands and feet, limb numbness, and rheumatic symptoms [107,128,129].

Notably, its primary active ingredient, gastrodin, has demonstrated favorable outcomes in the management of OP. Previous studies [105,130] have revealed that gastrodin (1 μM and 5 μM), by upregulating the Nrf2/kelch like ECH associated protein 1 (KEAP1) antioxidant pathway—encompassing Nrf2, HO-1, and nicotinamide adenine dinucleotide phosphate (NADPH)—substantially diminishes oxidative stress in dexamethasone-induced MC3T3-E1 cells and mitochondria, enhances osteoblast activity, promotes the expression of osteogenesis-related markers (such as Runx2, Osx, BMP-2, and osteocalcin (OCN)), and augments ALP activity and osteogenic mineralization capacity. Furthermore, other findings suggest that gastrodin (1 mg/kg/d and 5 mg/kg/d) alleviated glucocorticoid-induced OP in rats by protecting osteoblasts via the Nrf2 regulated mitochondrial and ER stress-related signaling pathways [106].

#### 4.1.3. Epimedium Folium

Epimedium folium is the dried leaves of *Epimedium brevicornu* Maxim., *Epimedium sagittatum* (Sieb.et Zucc.) Maxim., *Epimedium pubescens* Maxim., or *Epimedium koreanum* Nakai. of berberidaceae plant. This herbal remedy is highly valued in TCM for its ability to nourish kidney yang, fortify bones and muscles, and alleviate rheumatic conditions [131]. It is frequently prescribed to address symptoms such as kidney yang deficiency, impotence, spermatorrhea, muscular and bony laxity, rheumatic arthralgia, numbness, and muscular contractions [132,133].

Notably, icariin, its primary active constituent, has exhibited promising potential in the treatment of OP [134,135]. Previous research [136] demonstrated that ICA (100 mg/kg/d) forestalls ferroptosis-induced mitochondrial membrane potential dysfunction and ROS production, bolsters osteoblast survival, and reverses the suppression of Runx2, ALP, and OPG expression caused by ferroptosis. ICA also hinders the differentiation and function of osteoclasts and lessens iron accumulation in bone marrow, indicating its potential as a natural resource for developing therapeutics against ferroptosis-induced OP by modulating iron metabolism. Ferroptosis can impede the development and autophagy of osteoblast progenitor cells by inhibiting the PI3K/Akt/mTOR signaling pathway [137]. ICA (1 μM) can alleviate mitochondrial dysfunction and ROS production induced by iron overload in bone marrow mesenchymal stem cells (BMSCs) by activating the PI3K/AKT/mTOR pathway and inhibiting the MAPK pathways, thus facilitating the continued differentiation of mesenchymal stem cells into osteoblasts [138]. Moreover, XIANG et al. demonstrated that ICA (50 mg/kg/d and 150 mg/kg/d) could promote osteoporotic fracture healing by inhibiting osteoblast ferroptosis through activation of the antioxidant Nrf2/HO-1 signaling pathway in OVX rats [108].

#### 4.1.4. Scutellariae Radix

Scutellariae radix, derived from the dried root of th*e Scutellaria baicalensis* Georgi of labiate plant, is an herbal remedy esteemed in TCM for its multifaceted benefits. It effectively clears heat and dries dampness, purges excessive fire and detoxifies the body, stops bleeding, and promotes the relaxation of uterine muscles. Its applications span a wide range of conditions, including damp-warm syndromes, summer-heat dampness, chest tightness accompanied by vomiting, damp-heat congestion leading to diarrhea, jaundice, lung heat with coughing, high fever coupled with thirst, blood heat-induced vomiting, carbuncles, sore toxins, and fetal irritability, among others [139,140].

Notably, baicalein (BN), an active ingredient isolated from *Scutellaria baicalensis*, has shown promise in the treatment of OP [141], as evidenced by previous research studies [109] showed that BN (10 μM) enhances the osteogenic differentiation of glucocorticoid- induced MC3T3-E1 cells by inhibiting AKT and promoting the expression of forkhead box O1 (FoXO1), thereby inhibiting OP through the AKT/FoXO1 signaling pathway.

#### 4.1.5. Drynaria Rhizoma

Drynaria rhizoma, derived from the dried rhizome of the *Drynaria fortunei* (Kunze) J.Sm. of polypodiaceae plants, is a herbal remedy recognized in TCM for its diverse the treatment of orthopedic related diseases [142,143]. It is noted for its ability to alleviate pain, nourish the kidneys, strengthen bones, and eliminate external wind and spots when applied topically. In traditional practice, it is frequently utilized in the treatment of conditions such as flaccidity, tendon and bone fractures, kidney deficiency with accompanying low back pain, atrophy of tendons and bones, tinnitus and deafness, and tooth loosening. For external use, it is applied to treat alopecia areata and vitiligo, among other skin disorders [144].

Notably, naringenin, an efficacious active ingredient in Drynaria rhizoma, has demonstrated potential in treating OP by modulating ferroptosis. The results of cell experiments [110] found that 10 μM and 20 μM naringenin (NAR) alleviates cell viability, apoptosis, and MMP damage induced by the co-culture of ferric ammonium citrate and IL-1β, increases MDA levels, reduces the expression of MMP 3, MMP 13, and Bax, and restores the expression of type II collagen (Col II). NAR also exhibits a slight reduction in iron accumulation, alleviates the accumulation of ROS and lipid peroxidation in chondrocytes, upregulates the levels of Nrf2 and HO-1, and effectively improves cartilage damage and subchondral bone proliferation.

### 4.2. Chinese Herbal Remedies

#### 4.2.1. Erxian Decoction

EXD is a classical traditional Chinese herbal formula from *Curculigo orchioides* Gaertn., *Epimedium brevicornu* Maxim, *Angelica sinensis*(Oliv.)Diels, *Morinda officinalis* How, *Phellodendron chinense* Schneid., and *Anemarrhena asphodeloides* Bge., which has been used to treat OP for several decades [145,146]. In traditional practice, it possesses the effects of warming kidney yang, nourishing kidney essence, and clearing kidney fire. It can be used for treating menopause syndrome, hypertension, amenorrhea, and other chronic diseases characterized by deficiency of both kidney yin and yang with disturbance by deficient fire. The herbs such as Curculiginis rhizoma, Epimedium folium, and Morindae officinalis radix in EXD have the function of tonifying kidney yang and strengthening the kidneys, which can enhance kidney function, promote bone marrow hematopoiesis, strengthen bones, and help improve the microstructure of bones and increase bone density [147,148,149]. Additionally, the blood-nourishing herbs like Angelica sinensis radix in EXD can warm and nourish the blood to replenish deficiencies, aiding in bone repair and alleviating pain symptoms [150,151]. The blood-activating and collateral-unblocking effects of Angelica sinensis radix can also improve microcirculation, further promoting bone health.

These effects may indirectly influence cellular processes such as ferroptosis by improving the bone microenvironment and reducing damage and death of bone cells [152,153]. Previous research findings [111] demonstrated that the EXD could stimulate the expression of Nrf2 in osteoblasts under HG conditions, reduce the levels of ROS, and ameliorate the oxidative stress injury to osteoblasts caused by elevated glucose levels. Moreover, other evidence suggests that EXD (9 g/kg/d) can improve bone loss induced by ovariectomy by regulating lipid metabolism, fatty acid metabolism, and the IGF1/PI3K/AKT pathway [112].

#### 4.2.2. Bugu Shengsui Capsule

Bugu Shengsui Capsule represents an effective Chinese medical compound formula with an array of herbs including *Eucommia ulmoides* Oliv., *Psoralea corylifolia* L., *Cervus nippon* Temminck., *Commiphora myrrha* Engl., which is applied to treat OP in clinical practice. It can effectively increase bone mineral density (BMD), serum calcitonin, luteinizing hormone, and calcium in OP patients, with total 82% efficiency [154]. Bugu Shengsui Capsule can also improve blood circulation disorders in osteoporotic model rats.

Xu et al. evaluated the role of Bugu Shengsui Capsule in the animal model of orchiectomy. Quantitative proteomics analysis combined with RNA interference experiments showed that Bugu Shengsui Capsule (2.94, 5.87, and 11.74 g/kg/d) could promote osteogenesis through the PI3K-AKT pathway [113]. Meanwhile, an in vivo study with senescence-accelerated mice prone 6 (SAMP1) showed that Bugu Shengsui Capsule has a positive effect on osteoblast differentiation and bone formation. Such responsiveness may predominantly be associated with the ERK/Smad signaling pathways, which suggests that Bugu Shengsui Capsule (10.1, 20.32 and 40.64 g/kg/d) is an effective drug for the prevention and treatment of OP [114].

#### 4.2.3. Compound Lurong Jiangu Capsule

Compound Lurong Jiangu Capsule is composed of *Cervus nippon* Temminck, Polygon multiflori radix praeparata, *Chinemys reevesii* (Gray), *Eucommia ulmoides* Oliv., *Placenta* Hominis, *Angelica sinensis* (Oliv.) Diels, *Panax notoginseng* (Burk.) F. H. Chen, *Hirudo nipponica* Whitman and *Amomum villosum* Lour. It has the effect of tonifying kidney and strengthening bone, activating blood and relieving pain. It is used to treat OP, which belongs to liver and kidney deficiency syndrome. The symptoms include low back pain, soreness and weakness of waist and knees, foot root pain, dizziness, deafness and tinnitus. Previous literature studies have shown the effect of tonifying kidney and strengthening bone, harmonizing qi and blood, which has a significant effect on OP [155]. Studies indicate that this compound (50,100 and 200 μg/mL) can substantially decrease oxidative damage in osteoblasts induced by hydrogen peroxide by activating the Nrf2/HO-1 signaling pathway and upregulating the expression of osteogenic transcription factors such as Runx2 and Osx, offering new perspectives for treating oxidative damage-induced OP [115].

#### 4.2.4. Xianling Gubao Capsule

Xianling Gubao Capsule is composed of five Chinese herbal medicines: Epimedium brevicornum, *Dipsacus asper* Wall. ex-Henry., *Psoralea corylifolia* L., *Rehmannia glutinosa* Libosch., *Salvia miltiorrhiza* Bge., and *Anemarrhena asphodeloides* Bge. It has been a mainstay in the treatment of OP, osteoarthritis, aseptic OP, and PMOP for over two decades [156,157,158]. Notably, Chen et al. demonstrated that the capsule significantly reduces HG-induced apoptosis in human osteoblast-like cells (MG63 cells) and modulates the expression of proteins associated with mitochondrial apoptosis [116]. This finding indicates that Xianling Gubao Capsule (200 mg/mL) may protect osteoblasts from HG-induced cell death and enhance bone formation, offering a promising therapeutic approach for bone health.

#### 4.2.5. Qing’e Pill

Qing’e Pill (QEP) is composed of *Eucommia ulmoides* Oliv., *Psoralea corylifolia* L., *Allium sativum* L. and *Juglans regia* L., which has the effect of tonifying kidney and strengthening waist, and it has been shown to play an anti-OP role by regulating the bone metabolism-related factors, promoting the bone formation, inhibiting the bone absorption, and playing an active role in the prevention and treatment of OP [159,160,161]. This prescription is commonly used in clinical treatment of kidney deficiency low back pain, sit-ups, knee weakness, and orthopedic related diseases. In the prescription, Eucommiae cortex has the function of tonifying liver and kidney, strengthening waist and knees, and strengthening tendons and bones, which is the monarch drug. Psoraleae fructus can tonify kidney yang, as a ministerial medicine. The Juglandis semen can nourish the kidney and solidify the essence. The Allii sativi bulbus can alleviate the cold, the stagnation of the collaterals, the strong waist pain, acting in combination as an adjuvant drug. In previous studies, Hao et al. utilized network pharmacology-molecular docking technology combined with high-performance liquid chromatography to predict the potential target genes of QEP (4.5 g/kg/d) for OP and ferroptosis. Subsequent validation using an OVX rat model and HFOB 1.19 cells confirmed that QEP can improve OP and inhibit ferroptosis through AKT/PI3K pathways [117].

#### 4.2.6. Zuogui Pill

The Zuogui Pill, a clinically approved formulation in China, is composed of Rehmannia radix praeparata., *Dioscorea opposita* Thunb., *Lycium barbarum* L., *Cornus officinalis* Sieb. et Zucc., *Cyathula officinalis* Kuan., *Cuscuta australis* R.Br., Cervi cornus colla. and Testudinis Plastrum Colla. It has the effect of nourishing yin and kidney and nourishing essence and blood. Traditional medicine is often used in the treatment of deficiency of kidney water, but it cannot provide the nutrition and defense and leads gradually to weakness, virtual heat exchanges, spontaneous sweating, residual stranguria, deafness, dry mouth and dry tongue, and lumbar acid leg soft. It has been a quintessential prescription in TCM for nourishing yin and tonifying the kidneys and is commonly used for treating kidney yin deficiency-type OP [162]. Previous research [118] found that the intervention with the Zuogui Pill (0.96 g/100 g/d) in model rats increased the expression of osteoprotegerin (OPG) and decreased the expression of receptor activator of nuclear factor-κB ligand (RANKL), indicating that the Zuogui Pill may modulate cellular ferroptosis through the OPG/RANKL signaling pathway, thereby exerting an anti-OP effect.

### 4.3. Limitations and Challenges of Chinese Herbal Interventions in Treating OP via Ferroptosis

While Chinese herbs have demonstrated potential in treating OP within certain contexts, their application faces multiple constraints and challenges. Currently, there is a lack of high-quality, large-scale evidence-based medical evidence to support their efficacy, as most studies remain confined to animal experiments or small-scale clinical trials, resulting in limited international recognition. The complex composition of herbal formulas and incomplete understanding of their mechanisms of action often lead to inconsistent therapeutic outcomes, batch-to-batch variability, and difficulties in standardization. Additionally, the slow onset of effects and prolonged treatment duration reduce patient compliance, particularly for high-risk individuals requiring rapid intervention in acute fracture scenarios. Certain Chinese herbs carry potential hepatorenal toxicity, and long-term use may cause drug accumulation-related damage, while risks of interactions with Western medications (e.g., anticoagulants, hormones) remain understudied. TCM emphasizes syndrome differentiation and treatment but accurately identifying patterns such as “kidney deficiency” or “blood stasis” poses challenges for non-specialized practitioners, with misuse potentially exacerbating conditions. Variations in raw herb quality due to differences in cultivation regions and processing techniques, coupled with regulatory loopholes, enable the circulation of substandard products. Although single-herb interventions have shown significant efficacy in non-clinical OP studies, their clinical application remains rare. Overall, herbal therapies require professional guidance alongside Western medical approaches, and future efforts must prioritize enhanced basic research, standardized production, and rigorous evidence-based validation to clarify their scientific role.

## 5. Conclusions

In recent years, substantial advancements have been achieved in investigating the role of ferroptosis in the pathogenesis of OP and its intervention using Chinese herbs. Ferroptosis, a regulated form of programmed cell death, plays a pivotal role in the initiation and progression of numerous diseases. As research delves deeper, compelling evidence suggests that modulating ferroptosis can emerge as a potent therapeutic strategy for disease management. In the pathological milieu of OP, the equilibrium of iron ions is frequently disrupted, leading to either iron overload or deficiency, which subsequently impacts normal iron metabolism. These perturbations further influence the metabolism and functionality of bone cells, thereby exacerbating the progression of OP. Moreover, ferroptosis is intricately linked to oxidative stress responses within bone cells, while in regard to OP, bone cells are provoked by a multitude of internal and external stimuli, resulting in the generation of substantial quantities of ROS. These ROS assault proteins and lipids within bone cells, leading to cellular dysfunction. Iron ions, as crucial participants in ROS production, further intensify oxidative stress responses and facilitate the occurrence of ferroptosis.

OP manifests in various syndromes, and this article primarily summarizes the interplay between DOP, GIOP, PMOP, and ferroptosis. In DOP, hyperglycemic conditions and the accumulation of advanced glycation end products in collagen evoke oxidative stress, resulting in decreased bone formation and ultimately culminating in OP. Glucocorticoids, which are commonly prescribed for a range of inflammatory and autoimmune disorders, also exacerbate the progression of OP by inducing ferroptosis in osteoblasts and enhancing bone resorption. PMOP is characterized by a decline in estrogen levels, which leads to iron accumulation in bone tissue and triggers ferroptosis in bone cells, thereby diminishing bone mineral density and augmenting the risk of fractures.

Fortunately, several bioactive components derived from TCM have demonstrated definitive therapeutic efficacy in OP intervention through ferroptosis regulation. These include quercetin, cortex polysaccharides and AU isolated from Eucommia ulmoides, gastrodin extracted from Gastrodia elata, ICA obtained from Epimedium folium, baicalin purified from Scutellaria baicalensis, and naringenin identified in Psoralea corylifolia. Furthermore, classical Chinese herbal formulations such as Bugu Shengsui Formula, Compound Lurong Jiangu Capsule, Xianling Gubao Capsule, Qing’e Pill, and Zuogui Pill have also exhibited significant therapeutic benefits in the treatment of OP. By precisely targeting the ferroptosis process, regulating iron metabolism, and employing antioxidant interventions, these herbs can effectively ameliorate OP symptoms, offering novel insights and methodologies for the treatment of this condition. Additionally, Chinese herbs exhibit unique advantages in modulating these processes, presenting a promising approach to managing OP.

It is worth noting that despite this article’s review of the role of ferroptosis in the pathogenesis of OP and the therapeutic potential of Chinese herbal interventions, several limitations and challenges remain. First, although Chinese herbs show efficacy in modulating ferroptosis, their bioactive components often act through multiple pathways, complicating the identification of specific molecular targets. Additionally, the dual role of iron in bone homeostasis poses a therapeutic dilemma: interventions aiming to suppress ferroptosis must balance iron chelation or antioxidant strategies without disrupting physiological iron-dependent processes. This complexity is compounded by individual variability in iron metabolism, which may influence treatment responsiveness. Addressing these gaps requires multidisciplinary efforts to refine mechanistic insights, optimize herbal formulations, and conduct rigorous clinical validations to translate ferroptosis-targeted strategies into safe, effective, and personalized OP therapies.

## Figures and Tables

**Table 1:** Mechanism of action of Chinese herb regulating ferroptosis.

Chinese Herb	Source	Medicament Portions	Traditional Use	Experimental Model	Signaling Pathway	Mechanisms	Ref.
Quercetin	*Eucommia ulmoides* Oliv.	Bark	Tonifying liver and kidney, strong bones, tocolysis	MC3T3-E1 cells, Iron overload mice	Nrf2/HO-1	ROS↓, ALP↑, Runx2↑, Osx↑, Caspase3↓, Bax↓, Nrf2↑	[101]
Eucommia cortex polysaccharide				GIOP mice	ERK/JNK/Nrf2	pERK↑, pJNK↓, Nrf2↑, NQO-1↑	[102,103]
Aucubin				BMSCs cells, OVX rats	BMP2/SMADs	ROS↓, Fe^2+^↓, MDA↓, SOD↑, GPX4↑	[104]
Gastrodine	*Gastrodia elata* Bl.	Tuber	Xifeng antispasmodic, calm the liver Yang, Qufeng Tongluo	MC3T3-E1 cells	Nrf2/KEAPl	ROS↓, Runx2↑, Osx↑, BMP2↓, OCN↓	[105]
				GIOP rats	Nrf2/KEAP1	ROS↓, Nrf2↑	[106]
Icariin	*Epimedium brevicornu* Maxim.	Leaves	Tonify kidney yang, strong bones, dispel rheumatism	BMSCs cells	PI3K/AKT/mTOR,MAPK	ROS↓, Runx2↑, Osx↑, β-catenin↑, pPI3K↓, pAkT↓, pmTOR↓	[107]
				OVX rats	Nrf2/HO-1	Nrf2↑, NQO-1↑, HO-1↑, Runx2↑, ALP↑, OPG↑, OCN↑, GPX4↑, BAX↓	[108]
Baicalein	*Scutellaria baicalensis* Georgi	Root	Clearing heat and drying dampness, purging fire detoxification, hemostasis, tocolysis	MC3T3-E1 cells	AKT/FoXO1	AKT↓, FoXO1↑	[109]
Naringenin	*Drynaria fortune* (Kunze) J.Sm.	Rhizome	Healing pain, tonifying kidney and strengthening bones	Iron overload mice	Nrf2/HO-1	ROS↓, MDA↓, MMP3↓, MMP13↓, Bax↓, HO-1↑, Nrf2↑	[110]

Note:”↑” represent upward adjustments, ”↓” represent downward adjustments.

**Table 2:** Mechanism of action of Chinese herbal remedies regulating ferroptosis.

Chinese Herbal Remedy	Composition	Experimental Model	Signaling Pathway	Mechanisms	Ref.
Erxian Decoction	Flavonoids (icariin, curculigoside), saponins (timosaponin, morin), alkaloids (berberine) and polysaccharides.	Human primary bone cells	RANKL/OPG	RANKL/OPG↓, Nrf2↑	[111]
		OVX rats	IGF1/PI3K/AKT	IGF1R↓, PI3K↓, AKT↓	[112]
Bugu Shengsui Capsule	Flavonoids (icariin, psoralen), saponins (timosaponin, astragaloside), alkaloids (berberine) and polysaccharides (angelica polysaccharide).	OVX rats	Oxidative stress and iron accumulation	MDA↓, Ferritin↓, SOD↑, Hepcidin↑,	[113]
		SAMP6 mice	ERK/Smad	ALP↑, RUNX2↑, ERK↑, Smad↑	[114]
Compound Lurong Jiangu Capsule	Velvet antler polypeptide, eucommia ulmoides iridoid, angelica ferulic acid, notoginsenoside and tortoise shell mineral.	BMSCs cells	Nrf2/HO-1	ROS↓, ALP↑, HO-1↑, Nrf2↑	[115]
Xianling Gubao Capsule	Flavonoids (icariin), saponins (asperosaponin VI, timosaponin), coumarins (psoralen, isopsoralen), phenolic acids (salvianolic acid), diterpenoids (tanshinone), polysaccharides (rehmannia polysaccharide).	MG63 cells	OPG/OPGL, PI3K/Akt	OCN↑, OPN↑, RUNX2↑, OPG↑, OPGL↓, pAKT↓, p70S6K↓	[116]
Qing’e Pill	Coumarins (psoralen, isopsoralen), iridoids (eucommia gum), polyunsaturated fatty acids (linoleic acid), organic sulfur (allicin).	OVX rats	ATM, PI3K/AKT	GPX4↑, Xct↑, pPI3K↓, pAKT↓, BMP2↓, MDA↓	[117]
Zuogui Pill	Iridoids (catalpol, cornusoside), polysaccharides (yam polysaccharide), flavonoids (dodder flavonoids), saponins (dioscin), antler glue, tortoise shell glue.	OVX rats	OPG/RANKL	Ferric ion↓, Hepcidin↑, OPG↓, RANKL↑	[118]

Note:”↑” represent upward adjustments, ”↓” represent downward adjustments.

## References

[1] Lyu Z., Hu Y., Guo Y., Liu D. (2023). Modulation of Bone Remodeling by the Gut Microbiota: A New Therapy for Osteoporosis. Bone Res..

[2] Adolpho L.F., Gomes M.P.O., Freitas G.P., Bighetti-Trevisan R.L., Ramos J.I.R., Campeoti G.H., Zatta G.C., Almeida A.L.G., Tarone A.G., Marostica-Junior M.R. (2024). Jaboticaba Peel Extract Attenuates Ovariectomy-Induced Bone Loss by Preserving Osteoblast Activity. Biology.

[3] Lindsay R., Aitken J.M., Anderson L.B., Hart D.M., Macdonald E.B., Clarke A.C. (1976). Long-Term Prevention of Postmenopausal Osteoporosis by Œstrogen. Lancet.

[4] Li H., Xiao Z., Quarles L.D., Li W. (2021). Osteoporosis: Mechanism, Molecular Target and Current Status on Drug Development. Curr. Med. Chem..

[5] Weaver C.M., Alexander D.D., Boushey C.J., Dawson-Hughes B., Lappe J.M., LeBoff M.S., Liu S., Looker A.C., Wallace T.C., Wang D.D. (2016). Calcium plus Vitamin D Supplementation and Risk of Fractures: An Updated Meta-Analysis from the National Osteoporosis Foundation. Osteoporos. Int..

[6] Drake M.T., Clarke B.L., Khosla S. (2008). Bisphosphonates: Mechanism of Action and Role in Clinical Practice. Mayo Clin. Proc..

[7] Sharpe M., Noble S., Spencer C.M. (2001). Alendronate. Drugs.

[8] Vahle J.L., Long G.G., Sandusky G., Westmore M., Ma Y.L., Sato M. (2004). Bone Neoplasms in F344 Rats Given Teriparatide [rhPTH(1-34)] Are Dependent on Duration of Treatment and Dose. Toxicol. Pathol..

[9] Yu T., Wang Z., You X., Zhou H., He W., Li B., Xia J., Zhu H., Zhao Y., Yu G. (2020). Resveratrol Promotes Osteogenesis and Alleviates Osteoporosis by Inhibiting P53. Aging.

[10] Bao J., Yan Y., Zuo D., Zhuo Z., Sun T., Lin H., Han Z., Zhao Z., Yu H. (2023). Iron Metabolism and Ferroptosis in Diabetic Bone Loss: From Mechanism to Therapy. Front. Nutr..

[11] Yu T., You X., Zhou H., He W., Li Z., Li B., Xia J., Zhu H., Zhao Y., Yu G. (2020). MiR-16-5p Regulates Postmenopausal Osteoporosis by Directly Targeting VEGFA. Aging.

[12] Wang D., Shen J., Wang Y., Cui H., Li Y., Zhou L., Li G., Wang Q., Feng X., Qin M. (2025). Mechanisms of Ferroptosis in Bone Disease: A New Target for Osteoporosis Treatment. Cell. Signal..

[13] Deeks E.D. (2018). Denosumab: A Review in Postmenopausal Osteoporosis. Drugs Aging.

[14] Nakamura T., Naguro I., Ichijo H. (2019). Iron Homeostasis and Iron-Regulated ROS in Cell Death, Senescence and Human Diseases. Biochim. Et Biophys. Acta (BBA)—Gen. Subj..

[15] van Swelm R.P.L., Wetzels J.F.M., Swinkels D.W. (2020). The Multifaceted Role of Iron in Renal Health and Disease. Nat. Rev. Nephrol..

[16] Cui H., Wang Y., Ma J., Zhou L., Li G., Li Y., Sun Y., Shen J., Ma T., Wang Q. (2024). Advances in Exosome Modulation of Ferroptosis for the Treatment of Orthopedic Diseases. Pathol. Res. Pract..

[17] Wang D. (2023). Progress in the Study of Ferroptosis in Cancer Treatment: State-of-the-Art. Chem.-Biol. Interact..

[18] Le Y., Zhang Z., Wang C., Lu D. (2021). Ferroptotic Cell Death: New Regulatory Mechanisms for Metabolic Diseases. Endocrine, Metab. Immune Disord. Drug Targets.

[19] Gao Z., Chen Z., Xiong Z., Liu X. (2022). Ferroptosis*—*A New Target of Osteoporosis. Phytother. Res..

[20] Mao C., Liu X., Zhang Y., Lei G., Yan Y., Lee H., Koppula P., Wu S., Zhuang L., Fang B. (2021). DHODH-Mediated Ferroptosis Defence Is a Targetable Vulnerability in Cancer. Nature.

[21] Wen S., Aki T., Unuma K., Uemura K. (2020). Chemically Induced Models of Parkinson’s Disease: History and Perspectives for the Involvement of Ferroptosis. Front. Cell. Neurosci..

[22] Chen B., Yan Y.-L., Liu C., Bo L., Li G.-F., Wang H., Xu Y.-J. (2014). Therapeutic Effect of Deferoxamine on Iron Overload-Induced Inhibition of Osteogenesis in a Zebrafish Model. Calcif. Tissue Int..

[23] Xia D., Wu J., Xing M., Wang Y., Zhang H., Xia Y., Zhou P., Xu S. (2019). Iron Overload Threatens the Growth of Osteoblast Cells via Inhibiting the PI3K/AKT/FOXO3a/DUSP14 Signaling Pathway. J. Cell. Physiol..

[24] Che J., Yang J., Zhao B., Zhang G., Wang L., Peng S., Shang P. (2020). The Effect of Abnormal Iron Metabolism on Osteoporosis. Biol. Trace Elem. Res..

[25] Baschant U., Rauner M., Balaian E., Weidner H., Roetto A., Platzbecker U., Hofbauer L.C. (2016). Wnt5a Is a Key Target for the Pro-Osteogenic Effects of Iron Chelation on Osteoblast Progenitors. Haematologica.

[26] Wu D., Wen X., Liu W., Hu H., Ye B., Zhou Y. (2018). Comparison of the Effects of Deferasirox, Deferoxamine, and Combination of Deferasirox and Deferoxamine on an Aplastic Anemia Mouse Model Complicated with Iron Overload. Drug Des. Dev. Ther..

[27] Wang J., Chen T., Gao F. (2024). Mechanism and Application Prospect of Ferroptosis Inhibitors in Improving Osteoporosis. Front. Endocrinol..

[28] Yao X., Huang Y., Li Y., Zhao W. (2021). [Salvianolic Acid B Promotes the Proliferation, Migration and Osteogenic Differentiation of Human Gingival Mesenchymal Stem Cells by Activating the PI3K/AKT Pathway]. Xi Bao Yu Fen Zi Mian Yi Xue Za Zhi = Chin. J. Cell. Mol. Immunol..

[29] Diaz M. (1998). Effect of Desferrioxamine and Deferiprone (L1) on the Proliferation of MG-63 Bone Cells and on Phosphatase Alkaline Activity. Nephrol. Dial. Transplant. Off. Publ. Eur. Dial. Transpl. Assoc.—Eur. Ren. Assoc..

[30] Messa E., Carturan S., Maffe C., Pautasso M., Bracco E., Roetto A., Messa F., Arruga F., Defilippi I., Rosso V. (2010). Deferasirox Is a Powerful NF-kappaB Inhibitor in Myelodysplastic Cells and in Leukemia Cell Lines Acting Independently from Cell Iron Deprivation by Chelation and Reactive Oxygen Species Scavenging. Haematologica.

[31] Guan H., Cao R., Zhao Y., Zhang J., Li H., Duan X., Li Y., Kong N., Tian R., Wang K. (2023). [Melatonin Promotes Osteogenesis of Bone Marrow Mesenchymal Stem Cells by Improving the Inflammatory State in Ovariectomized Rats]. Zhongguo Xiu Fu Chong Jian Wai Ke Za Zhi = Zhongguo Xiufu Chongjian Waike Zazhi = Chin. J. Reparative Reconstr. Surg..

[32] Jiang Z., Wang H., Qi G., Jiang C., Chen K., Yan Z. (2022). Iron Overload-induced Ferroptosis of Osteoblasts Inhibits Osteogenesis and Promotes Osteoporosis: An in Vitro and in Vivo Study. IUBMB Life.

[33] Xu P., Lin B., Deng X., Huang K., Zhang Y., Wang N. (2022). VDR Activation Attenuates Osteoblastic Ferroptosis and Senescence by Stimulating the Nrf2/GPX4 Pathway in Age-Related Osteoporosis. Free Radic. Biol. Med..

[34] Liu P., Wang W., Li Z., Li Y., Yu X., Tu J., Zhang Z. (2023). Ferroptosis: A New Regulatory Mechanism in Osteoporosis. Phytomedicine.

[35] Guo C., Huang Q., Wang Y., Yao Y., Li J., Chen J., Wu M., Zhang Z., E M., Qi H. (2023). Therapeutic Application of Natural Products: NAD+ Metabolism as Potential Target. Phytomedicine.

[36] Lim J.-M., Yoo H.J., Lee K.-W. (2022). High Molecular Weight Fucoidan Restores Intestinal Integrity by Regulating Inflammation and Tight Junction Loss Induced by Methylglyoxal-Derived Hydroimidazolone-1. Mar. Drugs.

[37] Wang Z., Xiong Y., Peng Y., Zhang X., Li S., Peng Y., Peng X., Zhuo L., Jiang W. (2023). Natural Product Evodiamine-Inspired Medicinal Chemistry: Anticancer Activity, Structural Optimization and Structure-Activity Relationship. Eur. J. Med. Chem..

[38] Gao M., Monian P., Quadri N., Ramasamy R., Jiang X. (2015). Glutaminolysis and Transferrin Regulate Ferroptosis. Mol. Cell.

[39] Tang D., Chen X., Kang R., Kroemer G. (2021). Ferroptosis: Molecular Mechanisms and Health Implications. Cell Res..

[40] Theil E.C. (2013). Ferritin: The Protein Nanocage and Iron Biomineral in Health and in Disease. Inorg. Chem..

[41] Wang F., Lv H., Zhao B., Zhou L., Wang S., Luo J., Liu J., Shang P. (2019). Iron and Leukemia: New Insights for Future Treatments. J. Exp. Clin. Cancer Res..

[42] Ajoolabady A., Aslkhodapasandhokmabad H., Libby P., Tuomilehto J., Lip G.Y.H., Penninger J.M., Richardson D.R., Tang D., Zhou H., Wang S. (2021). Ferritinophagy and Ferroptosis in the Management of Metabolic Diseases. Trends Endocrinol. Metab..

[43] Hou W., Xie Y., Song X., Sun X., Lotze M.T., Zeh H.J., Kang R., Tang D. (2016). Autophagy Promotes Ferroptosis by Degradation of Ferritin. Autophagy.

[44] Gao M., Monian P., Pan Q., Zhang W., Xiang J., Jiang X. (2016). Ferroptosis Is an Autophagic Cell Death Process. Cell Res..

[45] Stockwell B.R., Jiang X., Gu W. (2020). Emerging Mechanisms and Disease Relevance of Ferroptosis. Trends Cell Biol..

[46] Brown C.W., Amante J.J., Chhoy P., Elaimy A.L., Liu H., Zhu L.J., Baer C.E., Dixon S.J., Mercurio A.M. (2019). Prominin2 Drives Ferroptosis Resistance by Stimulating Iron Export. Dev. Cell.

[47] Cheng Y., Song Y., Chen H., Li Q., Gao Y., Lu G., Luo C. (2021). Ferroptosis Mediated by Lipid Reactive Oxygen Species: A Possible Causal Link of Neuroinflammation to Neurological Disorders. Oxidative Med. Cell. Longev..

[48] Sun Y., Yan C., He L., Xiang S., Wang P., Li Z., Chen Y., Zhao J., Yuan Y., Wang W. (2023). Inhibition of Ferroptosis through Regulating Neuronal Calcium Homeostasis: An Emerging Therapeutic Target for Alzheimer’s Disease. Ageing Res. Rev..

[49] Vitalakumar D., Sharma A., Flora S.J.S. (2021). Ferroptosis: A Potential Therapeutic Target for Neurodegenerative Diseases. J. Biochem. Mol. Tox.

[50] Tang M., Chen Z., Wu D., Chen L. (2018). Ferritinophagy/Ferroptosis: Iron-related Newcomers in Human Diseases. J. Cell. Physiol..

[51] Qiu Y., Cao Y., Cao W., Jia Y., Lu N. (2020). The Application of Ferroptosis in Diseases. Pharmacol. Res..

[52] Rogers J.T., Cahill C.M. (2020). Iron-Responsive-like Elements and Neurodegenerative Ferroptosis. Learn. Mem..

[53] Chen Y., Guo X., Zeng Y., Mo X., Hong S., He H., Li J., Fatima S., Liu Q. (2023). Oxidative Stress Induces Mitochondrial Iron Overload and Ferroptotic Cell Death. Sci. Rep..

[54] Chen G.-H., Song C.-C., Pantopoulos K., Wei X.-L., Zheng H., Luo Z. (2022). Mitochondrial Oxidative Stress Mediated Fe-Induced Ferroptosis via the NRF2-ARE Pathway. Free Radic. Biol. Med..

[55] Stockwell B.R., Friedmann Angeli J.P., Bayir H., Bush A.I., Conrad M., Dixon S.J., Fulda S., Gascón S., Hatzios S.K., Kagan V.E. (2017). Ferroptosis: A Regulated Cell Death Nexus Linking Metabolism, Redox Biology, and Disease. Cell.

[56] Liu L., Yang S., Wang H. (2021). α-Lipoic Acid Alleviates Ferroptosis in the MPP^+^-induced PC12 Cells via Activating the PI3K/Akt/Nrf2 Pathway. Cell Biol. Int..

[57] Li J., Li M., Ge Y., Chen J., Ma J., Wang C., Sun M., Wang L., Yao S., Yao C. (2022). β-Amyloid Protein Induces Mitophagy-Dependent Ferroptosis through the CD36/PINK/PARKIN Pathway Leading to Blood–Brain Barrier Destruction in Alzheimer’s Disease. Cell Biosci..

[58] Magtanong L., Ko P.-J., To M., Cao J.Y., Forcina G.C., Tarangelo A., Ward C.C., Cho K., Patti G.J., Nomura D.K. (2019). Exogenous Monounsaturated Fatty Acids Promote a Ferroptosis-Resistant Cell State. Cell Chem. Biol..

[59] Zou Y., Palte M.J., Deik A.A., Li H., Eaton J.K., Wang W., Tseng Y.-Y., Deasy R., Kost-Alimova M., Dančík V. (2019). A GPX4-Dependent Cancer Cell State Underlies the Clear-Cell Morphology and Confers Sensitivity to Ferroptosis. Nat. Commun..

[60] Cui J., Zhou Q., Yu M., Liu Y., Teng X., Gu X. (2022). 4-Tert-Butylphenol Triggers Common Carp Hepatocytes Ferroptosis via Oxidative Stress, Iron Overload, SLC7A11/GSH/GPX4 Axis, and ATF4/HSPA5/GPX4 Axis. Ecotoxicol. Environ. Saf..

[61] Weiland A., Wang Y., Wu W., Lan X., Han X., Li Q., Wang J. (2019). Ferroptosis and Its Role in Diverse Brain Diseases. Mol. Neurobiol..

[62] Yan H., Zou T., Tuo Q., Xu S., Li H., Belaidi A.A., Lei P. (2021). Ferroptosis: Mechanisms and Links with Diseases. Sig Transduct. Target. Ther..

[63] Yuan S., Wei C., Liu G., Zhang L., Li J., Li L., Cai S., Fang L. (2022). Sorafenib Attenuates Liver Fibrosis by Triggering Hepatic Stellate Cell Ferroptosis via HIF-1α/SLC7A11 Pathway. Cell Prolif..

[64] Zhou Y., Shen Y., Chen C., Sui X., Yang J., Wang L., Zhou J. (2019). The Crosstalk between Autophagy and Ferroptosis: What Can We Learn to Target Drug Resistance in Cancer?. Cancer Biol. Med..

[65] Du Y., Zhao Y., Sun Y., Xu A. (2023). Acid Sphingomyelinase Mediates Ferroptosis Induced by High Glucose via Autophagic Degradation of GPX4 in Type 2 Diabetic Osteoporosis. Mol. Med..

[66] Santana-Codina N., Gikandi A., Mancias J.D. (2021). The Role of NCOA4-Mediated Ferritinophagy in Ferroptosis. Adv. Exp. Med. Biol..

[67] Bai Y., Meng L., Han L., Jia Y., Zhao Y., Gao H., Kang R., Wang X., Tang D., Dai E. (2019). Lipid Storage and Lipophagy Regulates Ferroptosis. Biochem. Biophys. Res. Commun..

[68] Yang M., Chen P., Liu J., Zhu S., Kroemer G., Klionsky D.J., Lotze M.T., Zeh H.J., Kang R., Tang D. (2019). Clockophagy Is a Novel Selective Autophagy Process Favoring Ferroptosis. Sci. Adv..

[69] Yang R., Xu W., Zheng H., Zheng X., Li B., Jiang L., Jiang S. (2021). Exosomes Derived from Vascular Endothelial Cells Antagonize Glucocorticoid-induced Osteoporosis by Inhibiting Ferritinophagy with Resultant Limited Ferroptosis of Osteoblasts. J. Cell. Physiol..

[70] Anagnostis P., Paschou S.A., Gkekas N.N., Artzouchaltzi A.-M., Christou K., Stogiannou D., Vryonidou A., Potoupnis M., Goulis D.G. (2018). Efficacy of Anti-Osteoporotic Medications in Patients with Type 1 and 2 Diabetes Mellitus: A Systematic Review. Endocrine.

[71] Farr J.N., Drake M.T., Amin S., Melton L.J., McCready L.K., Khosla S. (2014). In Vivo Assessment of Bone Quality in Postmenopausal Women With Type 2 Diabetes. J. Bone Miner. Res. Off. J. Am. Soc. Bone Miner. Res..

[72] Epstein S., LeRoith D. (2008). Diabetes and Fragility Fractures—A Burgeoning Epidemic?. Bone.

[73] Napoli N., Chandran M., Pierroz D.D., Abrahamsen B., Schwartz A.V., Ferrari S.L., IOF Bone and Diabetes Working Group (2017). Mechanisms of Diabetes Mellitus-Induced Bone Fragility. Nat. Rev. Endocrinol..

[74] Eller-Vainicher C., Cairoli E., Grassi G., Grassi F., Catalano A., Merlotti D., Falchetti A., Gaudio A., Chiodini I., Gennari L. (2020). Pathophysiology and Management of Type 2 Diabetes Mellitus Bone Fragility. J. Diabetes Res..

[75] Ma H., Wang X., Zhang W., Li H., Zhao W., Sun J., Yang M. (2020). Melatonin Suppresses Ferroptosis Induced by High Glucose via Activation of the Nrf2/HO-1 Signaling Pathway in Type 2 Diabetic Osteoporosis. Oxidative Med. Cell. Longev..

[76] Yang Y., Lin Y., Wang M., Yuan K., Wang Q., Mu P., Du J., Yu Z., Yang S., Huang K. (2022). Targeting Ferroptosis Suppresses Osteocyte Glucolipotoxicity and Alleviates Diabetic Osteoporosis. Bone Res..

[77] Zhang Z., Ji C., Wang Y.-N., Liu S., Wang M., Xu X., Zhang D. (2022). Maresin1 Suppresses High-Glucose-Induced Ferroptosis in Osteoblasts via NRF2 Activation in Type 2 Diabetic Osteoporosis. Cells.

[78] Krümmel B., von Hanstein A.-S., Plötz T., Lenzen S., Mehmeti I. (2022). Differential Effects of Saturated and Unsaturated Free Fatty Acids on Ferroptosis in Rat β-Cells. J. Nutr. Biochem..

[79] He J., Li Z., Xia P., Shi A., FuChen X., Zhang J., Yu P. (2022). Ferroptosis and Ferritinophagy in Diabetes Complications. Mol. Metab..

[80] Xiong L., Zhou B., Young J.L., Wintergerst K., Cai L. (2022). Exposure to Low-Dose Cadmium Induces Testicular Ferroptosis. Ecotoxicol. Environ. Saf..

[81] Jin C., Tan K., Yao Z., Lin B., Zhang D., Chen W.-K., Mao S., Zhang W., Chen L., Lin Z. (2023). A Novel Anti-Osteoporosis Mechanism of VK2: Interfering with Ferroptosis via AMPK/SIRT1 Pathway in Type 2 Diabetic Osteoporosis. J. Agric. Food Chem..

[82] Xu C.-Y., Xu C., Xu Y.-N., Du S.-Q., Dai Z.-H., Jin S.-Q., Zheng G., Xie C.-L., Fang W.-L. (2024). Poliumoside Protects against Type 2 Diabetes-Related Osteoporosis by Suppressing Ferroptosis via Activation of the Nrf2/GPX4 Pathway. Phytomedicine.

[83] Chotiyarnwong P., McCloskey E.V. (2020). Pathogenesis of Glucocorticoid-Induced Osteoporosis and Options for Treatment. Nat. Rev. Endocrinol..

[84] Vandewalle J., Luypaert A., De Bosscher K., Libert C. (2018). Therapeutic Mechanisms of Glucocorticoids. Trends Endocrinol. Metab..

[85] Bunim J.J. (1955). Studies on Metacortandralone and Metacortandracin in Rheumatoid Arthritis; Antirheumatic Potency, Metabolic Effects, and Hormonal Properties. JAMA.

[86] Buttgereit F. (2020). Views on Glucocorticoid Therapy in Rheumatology: The Age of Convergence. Nat. Rev. Rheumatol..

[87] Rizzoli R., Biver E. (2015). Glucocorticoid-Induced Osteoporosis: Who to Treat with What Agent?. Nat. Rev. Rheumatol..

[88] Compston J. (2018). Glucocorticoid-Induced Osteoporosis: An Update. Endocrine.

[89] Van Staa T.P., Leufkens H.G.M., Abenhaim L., Zhang B., Cooper C. (2000). Use of Oral Corticosteroids and Risk of Fractures. J. Bone Miner. Res. Off. J. Am. Soc. Bone Miner. Res..

[90] Bersuker K., Hendricks J.M., Li Z., Magtanong L., Ford B., Tang P.H., Roberts M.A., Tong B., Maimone T.J., Zoncu R. (2019). The CoQ Oxidoreductase FSP1 Acts Parallel to GPX4 to Inhibit Ferroptosis. Nature.

[91] Lu J., Yang J., Zheng Y., Chen X., Fang S. (2019). Extracellular Vesicles from Endothelial Progenitor Cells Prevent Steroid-Induced Osteoporosis by Suppressing the Ferroptotic Pathway in Mouse Osteoblasts Based on Bioinformatics Evidence. Sci. Rep..

[92] Sun F., Zhou J., Liu Z., Jiang Z., Peng H. (2022). Dexamethasone Induces Ferroptosis via P53/SLC7A11/GPX4 Pathway in Glucocorticoid-Induced Osteonecrosis of the Femoral Head. Biochem. Biophys. Res. Commun..

[93] Miao W., He L., Zhang Y., Zhu X., Jiang Y., Liu P., Zhang T., Li C. (2022). Ferroptosis Is Partially Responsible for Dexamethasone-Induced T Cell Ablation, but Not Osteoporosis in Larval Zebrafish. Ecotoxicol. Environ. Saf..

[94] Li M., Yang N., Hao L., Zhou W., Li L., Liu L., Yang F., Xu L., Yao G., Zhu C. (2022). Melatonin Inhibits the Ferroptosis Pathway in Rat Bone Marrow Mesenchymal Stem Cells by Activating the PI3K/AKT/mTOR Signaling Axis to Attenuate Steroid-Induced Osteoporosis. Oxidative Med. Cell. Longev..

[95] D’Amelio P., Cristofaro M.A., Tamone C., Morra E., Di Bella S., Isaia G., Grimaldi A., Gennero L., Gariboldi A., Ponzetto A. (2008). Role of Iron Metabolism and Oxidative Damage in Postmenopausal Bone Loss. Bone.

[96] Okyay E., Ertugrul C., Acar B., Sisman A.R., Onvural B., Ozaksoy D. (2013). Comparative Evaluation of Serum Levels of Main Minerals and Postmenopausal Osteoporosis. Maturitas.

[97] Cai H., Zhang H., He W., Zhang H. (2023). Iron Accumulation and Its Impact on Osteoporotic Fractures in Postmenopausal Women. J. Zhejiang Univ. Sci. B.

[98] Lan C., Zhou X., Shen X., Lin Y., Chen X., Lin J., Zhang Y., Zheng L., Yan S. (2024). Suppression of IRF9 Promotes Osteoclast Differentiation by Decreased Ferroptosis via STAT3 Activation. Inflammation.

[99] Xue C., Luo H., Wang L., Deng Q., Kui W., Da W., Chen L., Liu S., Xue Y., Yang J. (2023). Aconine Attenuates Osteoclast-Mediated Bone Resorption and Ferroptosis to Improve Osteoporosis via Inhibiting NF-κB Signaling. Front. Endocrinol..

[100] Jiang Z., Qi G., He X., Yu Y., Cao Y., Zhang C., Zou W., Yuan H. (2024). Ferroptosis in Osteocytes as a Target for Protection Against Postmenopausal Osteoporosis. Adv. Sci..

[101] Xiao J., Zhang G., Chen B., He Q., Mai J., Chen W., Pan Z., Yang J., Li J., Ma Y. (2023). Quercetin Protects against Iron Overload-Induced Osteoporosis through Activating the Nrf2/HO-1 Pathway. Life Sci..

[102] Song J., Zhang Y., Zhu Y., Jin X., Li L., Wang C., Zhou Y., Li Y., Wang D., Hu M. (2023). Structural Characterization and Anti-Osteoporosis Effects of Polysaccharide Purified from Eucommia Ulmoides Oliver Cortex Based on Its Modulation on Bone Metabolism. Carbohydr. Polym..

[103] Song J., Zhang Y., Jin X., Zhu Y., Li Y., Hu M. (2024). Eucommia Ulmoides Oliver Polysaccharide Alleviates Glucocorticoid-Induced Osteoporosis by Stimulating Bone Formation via ERK/BMP-2/SMAD Signaling. Sci. Rep..

[104] Zheng Y., Sun R., Yang H., Gu T., Han M., Yu C., Chen P., Zhang J., Jiang T., Ding Y. (2025). Aucubin Promotes BMSCs Proliferation and Differentiation of Postmenopausal Osteoporosis Patients by Regulating Ferroptosis and BMP2 Signalling. J. Cell. Mol. Medi.

[105] Liu S., Fang T., Yang L., Chen Z., Mu S., Fu Q. (2018). Gastrodin Protects MC3T3-E1 Osteoblasts from Dexamethasone-Induced Cellular Dysfunction and Promotes Bone Formation via Induction of the NRF2 Signaling Pathway. Int. J. Mol. Med..

[106] Liu S., Zhou L., Yang L., Mu S., Fang T., Fu Q. (2018). Gastrodin Alleviates Glucocorticoid Induced Osteoporosis in Rats via Activating the Nrf2 Signaling Pathways. Oncotarget.

[107] Su Z., Yang Y., Chen S., Tang Z., Xu H., Su Z., Yang Y., Chen S., Tang Z., Xu H. (2023). The Processing Methods, Phytochemistry and Pharmacology of Gastrodia Elata Bl.: A Comprehensive Review. J. Ethnopharmacol..

[108] Xiang S., Zhao L., Tang C., Ling L., Xie C., Shi Y., Liu W., Li X., Cao Y. (2024). Icariin Inhibits Osteoblast Ferroptosis via Nrf2/HO-1 Signaling and Enhances Healing of Osteoporotic Fractures. Eur. J. Pharmacol..

[109] Cai P., Lu Y., Yin Z., Wang X., Zhou X., Li Z. (2021). Baicalein Ameliorates Osteoporosis via AKT/FOXO1 Signaling. Aging.

[110] Pan Z., He Q., Zeng J., Li S., Li M., Chen B., Yang J., Xiao J., Zeng C., Luo H. (2022). Naringenin Protects against Iron Overload-Induced Osteoarthritis by Suppressing Oxidative Stress. Phytomedicine.

[111] Pathak J.L., Bravenboer N., Luyten F.P., Verschueren P., Lems W.F., Klein-Nulend J., Bakker A.D. (2015). Mechanical Loading Reduces Inflammation-Induced Human Osteocyte-to-Osteoclast Communication. Calcif. Tissue Int..

[112] Ma Y., Hu J., Song C., Li P., Cheng Y., Wang Y., Liu H., Chen Y., Zhang Z., Ma Y. (2023). Er-Xian Decoction Attenuates Ovariectomy-Induced Osteoporosis by Modulating Fatty Acid Metabolism and IGF1/PI3K/AKT Signaling Pathway. J. Ethnopharmacol..

[113] Wei X., Qi B., Ma R., Zhang Y., Liu N., Fang S., Zhu Y., Xie Y., Dai J., Zhu L. (2022). Quantitative Proteomics Revealed the Pharmacodynamic Network of Bugu Shengsui Decoction Promoting Osteoblast Proliferation. Front. Endocrinol..

[114] Liu N., Qi B., Zhang Y., Fang S., Sun C., Li Q., Wei X. (2022). Bu-Gu-Sheng-Sui Decoction Promotes Osteogenesis via Activating the ERK/Smad Signaling Pathways. Front. Pharmacol..

[115] Jin W., Zhu X., Yao F., Xu X., Chen X., Luo Z., Zhao D., Li X., Leng X., Sun L. (2020). Cytoprotective Effect of Fufang Lurong Jiangu Capsule against Hydrogen Peroxide-Induced Oxidative Stress in Bone Marrow Stromal Cell-Derived Osteoblasts through the Nrf2/HO-1 Signaling Pathway. Biomed. Pharmacother..

[116] Chen X., Li Y., Zhang Z., Chen L., Liu Y., Huang S., Zhang X. (2022). Xianling Gubao Attenuates High Glucose-Induced Bone Metabolism Disorder in MG63 Osteoblast-like Cells. PLoS ONE.

[117] Hao J., Bei J., Li Z., Han M., Ma B., Ma P., Zhou X. (2022). Qing’e Pill Inhibits Osteoblast Ferroptosis via ATM Serine/Threonine Kinase (ATM) and the PI3K/AKT Pathway in Primary Osteoporosis. Front. Pharmacol..

[118] Liu M., Wu J., Li Y., Wang S., Zhao H., Liu H., Pan J., Zhang Z., Wang W., Ju D. (2018). Effect of Zuogui Pill on Iron Overload in Osteoporosis Model Rats. Chin. J. Tradit. Chin. Med..

[119] Huang L., Lyu Q., Zheng W., Yang Q., Cao G., Huang L., Lyu Q., Zheng W., Yang Q., Cao G. (2021). Traditional Application and Modern Pharmacological Research of Eucommia Ulmoides Oliv. Chin. Med..

[120] He X., Wang J., Li M., Hao D., Yang Y., Zhang C., He R., Tao R., He X., Wang J. (2014). Eucommia Ulmoides Oliv.: Ethnopharmacology, Phytochemistry and Pharmacology of an Important Traditional Chinese Medicine. J. Ethnopharmacol..

[121] Sun Y., Zhang Y., Sun M., Gao W., He Y., Wang Y., Yang B., Kuang H., Sun Y., Zhang Y. (2024). Advances in Eucommia Ulmoides Polysaccharides: Extraction, Purification, Structure, Bioactivities and Applications. Front. Pharmacol..

[122] Zhao X., Qu Q., Zhang Y., Zhao P., Qiu J., Zhang X., Duan X., Song X., Zhao X., Qu Q. (2024). Research Progress of Eucommia Ulmoides Oliv and Predictive Analysis of Quality Markers Based on Network Pharmacology. CPB.

[123] Bao L., Sun Y., Wang J., Li W., Liu J., Li T., Liu Z., Bao L., Sun Y., Wang J. (2024). A Review of “Plant Gold” Eucommia Ulmoides Oliv.: A Medicinal and Food Homologous Plant with Economic Value and Prospect. Heliyon.

[124] Guan M., Pan D., Zhang M., Leng X., Yao B., Guan M., Pan D., Zhang M., Leng X., Yao B. (2021). The Aqueous Extract of Eucommia Leaves Promotes Proliferation, Differentiation, and Mineralization of Osteoblast-Like MC3T3-E1 Cells. Evid. Based Complement. Altern. Med..

[125] Pan Y., Niu Y., Li C., Zhai Y., Zhang R., Guo X., Mei Q., Pan Y., Niu Y., Li C. (2014). Du-Zhong (*Eucommia Ulmoides*) Prevents Disuse-Induced Osteoporosis in Hind Limb Suspension Rats. Am. J. Chin. Med..

[126] Wang T., Fan L., Feng S., Ding X., An X., Chen J., Wang M., Zhai X., Li Y. (2022). Network Pharmacology of Iridoid Glycosides from Eucommia Ulmoides Oliver against Osteoporosis. Sci. Rep..

[127] Zhang R., Liu Z.G., Li C., Hu S.J., Liu L., Wang J.P., Mei Q.B. (2009). Du-Zhong (Eucommia ulmoides Oliv.) Cortex Extract Prevent OVX-Induced Osteoporosis in Rats. Bone.

[128] Gong M., Lai F., Chen J., Li X., Chen Y., He Y., Gong M., Lai F., Chen J., Li X. (2024). Traditional Uses, Phytochemistry, Pharmacology, Applications, and Quality Control of Gastrodia Elata Blume: A Comprehensive Review. J. Ethnopharmacol..

[129] Zhan H.-D., Zhou H.-Y., Sui Y.-P., Du X.-L., Wang W., Dai L., Sui F., Huo H.-R., Jiang T.-L., Zhan H.-D. (2016). The Rhizome of Gastrodia Elata Blume*—*An Ethnopharmacological Review. J. Ethnopharmacol..

[130] Li Y., Li F. (2022). Mechanism and Prospect of Gastrodin in Osteoporosis, Bone Regeneration, and Osseointegration. Pharmaceuticals.

[131] Qian H., Wu D., Li C., Liu X., Han X., Peng Y., Zhang H., Zhao B., Zhao Y., Qian H. (2024). A Systematic Review of Traditional Uses, Phytochemistry, Pharmacology and Toxicity of Epimedium Koreanum Nakai. J. Ethnopharmacol..

[132] Cui J., Lin L., Hao F., Shi Z., Gao Y., Yang T., Yang C., Wu X., Gao R., Ru Y. (2024). Comprehensive Review of the Traditional Uses and the Potential Benefits of Epimedium Folium. Front. Pharmacol..

[133] Indran I.R., Liang R.L.Z., Min T.E., Yong E.-L., Indran I.R., Liang R.L.Z., Min T.E., Yong E.-L. (2016). Preclinical Studies and Clinical Evaluation of Compounds from the Genus Epimedium for Osteoporosis and Bone Health. Pharmacol. Ther..

[134] Si Y., Li Y., Gu K., Yin H., Ma Y., Si Y., Li Y., Gu K., Yin H., Ma Y. (2024). Icariin Ameliorates Osteoporosis in Ovariectomized Rats by Targeting Cullin 3/Nrf2/OH Pathway for Osteoclast Inhibition. Biomed. Pharmacother..

[135] Wang Z., Wang D., Yang D., Zhen W., Zhang J., Peng S., Wang Z., Wang D., Yang D., Zhen W. (2018). The Effect of Icariin on Bone Metabolism and Its Potential Clinical Application. Osteoporos. Int..

[136] Jing X., Du T., Chen K., Guo J., Xiang W., Yao X., Sun K., Ye Y., Guo F. (2019). Icariin Protects against Iron Overload-induced Bone Loss via Suppressing Oxidative Stress. J. Cell. Physiol..

[137] Cen W., Feng Y., Li S., Huang L., Zhang T., Zhang W., Kong W., Jiang J. (2018). Iron Overload Induces G1 Phase Arrest and Autophagy in Murine Preosteoblast Cells. J. Cell. Physiol..

[138] Yao X., Jing X., Guo J., Sun K., Deng Y., Zhang Y., Guo F., Ye Y. (2019). Icariin Protects Bone Marrow Mesenchymal Stem Cells Against Iron Overload Induced Dysfunction Through Mitochondrial Fusion and Fission, PI3K/AKT/mTOR and MAPK Pathways. Front. Pharmacol..

[139] Zhao T., Tang H., Xie L., Zheng Y., Ma Z., Sun Q., Li X., Zhao T., Tang H., Xie L. (2019). *Scutellaria Baicalensis* Georgi. (Lamiaceae): A Review of Its Traditional Uses, Botany, Phytochemistry, Pharmacology and Toxicology. J. Pharm. Pharmacol..

[140] Shen J., Li P., Liu S., Liu Q., Li Y., Sun Y., He C., Xiao P., Shen J., Li P. (2021). Traditional Uses, Ten-Years Research Progress on Phytochemistry and Pharmacology, and Clinical Studies of the Genus Scutellaria. J. Ethnopharmacol..

[141] Qin W., Shang Q., Shen G., Li B., Zhang P., Zhang Y., Zhao W., Chen H., Liu H., Xie B. (2024). Restoring Bone-Fat Equilibrium: Baicalin’s Impact on P38 MAPK Pathway for Treating Diabetic Osteoporosis. Biomed. Pharmacother..

[142] Lu L., Wang Z., Zhang H., Liu T., Fang H., Lu L., Wang Z., Zhang H., Liu T., Fang H. (2022). *Drynaria Fortunei* Improves Lipid Profiles of Elderly Patients with Postmenopausal Osteoporosis *via* Regulation of Notch1-NLRP3 Inflammasome-Mediated Inflammation. Gynecol. Endocrinol..

[143] Peng C.-H., Lin W.-Y., Li C.-Y., Dharini K.K., Chang C.-Y., Hong J.-T., Lin M.-D., Peng C.-H., Lin W.-Y., Li C.-Y. (2022). Gu Sui Bu (Drynaria fortunei J. Sm.) Antagonizes Glucocorticoid-Induced Mineralization Reduction in Zebrafish Larvae by Modulating the Activity of Osteoblasts and Osteoclasts. J. Ethnopharmacol..

[144] Yin F., Xiao L., Zhang Y. (2015). Research Progress on Drynaria Fortunei Naringin on Inflammation and Bone Activity. Zhongguo Gu Shang.

[145] Wang S.W., Cheung H.P., Tong Y., Lu J., Ng T.B., Zhang Y.B., Zhang Z.-J., Lee K.F., Lam J.K.W., Sze S.C.W. (2017). Steroidogenic Effect of Erxian Decoction for Relieving Menopause via the P-Akt/PKB Pathway in Vitro and in Vivo. J. Ethnopharmacol..

[146] Wang N., Xin H., Xu P., Yu Z., Shou D., Wang N., Xin H., Xu P., Yu Z., Shou D. (2019). Erxian Decoction Attenuates TNF-α Induced Osteoblast Apoptosis by Modulating the Akt/Nrf2/HO-1 Signaling Pathway. Front. Pharmacol..

[147] Wang J., Huang Y., Guo L., Li J., Zhou S. (2023). Molecular Mechanism of Achyranthis Bidentatae Radix and Morindae Officinalis Radix in Osteoporosis Therapy: An Investigation Based on Network Pharmacology, Molecular Docking, and Molecular Dynamics Simulations. Biochem. Biophys. Rep..

[148] Wang L., He Y., Han T., Zhao L., Lv L., He Y., Zhang Q., Xin H. (2017). Metabolites of Curculigoside in Rats and Their Antiosteoporotic Activities in Osteoblastic MC3T3-E1 Cells. Fitoterapia.

[149] Zhu F., Wang J., Ni Y., Yin W., Hou Q., Zhang Y., Yan S., Quan R. (2021). Curculigoside Protects against Titanium Particle-Induced Osteolysis through the Enhancement of Osteoblast Differentiation and Reduction of Osteoclast Formation. J. Immunol. Res..

[150] Li W., Cai Y., Ouyang Y., Li X., Shi X., Cao S., Huang Y., Wu H., Yang H. (2024). Quality Evaluation of Angelica Sinensis Radix Dispensing Granules by Integrating Microvascular Activity and Chemical Analysis. J. Ethnopharmacol..

[151] Wei W.-L., Zeng R., Gu C.-M., Qu Y., Huang L.-F. (2016). Angelica Sinensis in China-A Review of Botanical Profile, Ethnopharmacology, Phytochemistry and Chemical Analysis. J. Ethnopharmacol..

[152] Li J.-Y., Jia Y.-S., Chai L.-M., Mu X.-H., Ma S., Xu L., Wei X., Li J.-Y., Jia Y.-S., Chai L.-M. (2017). Effects of Chinese Herbal Formula Erxian Decoction for Treating Osteoporosis: A Systematic Review. Clin. Interv. Aging.

[153] Yang L., Fan L., Wang K., Chen Y., Liang L., Qin X., Lu A., Cao P., Yu B., Guan D. (2021). Analysis of Molecular Mechanism of Erxian Decoction in Treating Osteoporosis Based on Formula Optimization Model. Oxidative Med. Cell. Longev..

[154] Xie Y.M., Zhang F.Z., Zhou W.Q. (1997). Clinical Study of Bugu Shengsui Capsule in Treating Primary Osteoporosis with Kidney-Yang Deficiency Syndrome. Zhongguo Zhong Xi Yi Jie He Za Zhi Zhongguo Zhongxiyi Jiehe Zazhi = Chin. J. Integr. Tradit. West. Med..

[155] Zhou Y., Huang J., Cao W., Yu A., Li P., Liang J., Leng X., Jin J., Yu P., Liu J. (2024). The Therapeutic Mechanism of Compound Lurong Jiangu Capsule for the Treatment of Cadmium-Induced Osteoporosis: Network Pharmacology and Experimental Verification. Front. Endocrinol..

[156] Chen Q., Zeng J., Chen Y., Yang Y., Zhang T., Luo T., Huang H. (2019). Efficacy of Xianling Gubao Capsule in Treating Sarco-Osteopenia. Medicine.

[157] Li Z.-R., Cheng L.-M., Wang K.-Z., Yang N.-P., Yang S.-H., He W., Wang Y.-S., Wang Z.-M., Yang P., Liu X.-Z. (2018). Herbal Fufang Xian Ling Gu Bao Prevents Corticosteroid-Induced Osteonecrosis of the Femoral Head—A First Multicentre, Randomised, Double-Blind, Placebo-Controlled Clinical Trial. J. Orthop. Transl..

[158] Xiao J., Zhang G., Mai J., He Q., Chen W., Li J., Ma Y., Pan Z., Yang J., Li S. (2022). Bioinformatics Analysis Combined with Experimental Validation to Explore the Mechanism of XianLing GuBao Capsule against Osteoarthritis. J. Ethnopharmacol..

[159] Sun P., Zhang Y., Wei Z., Wang Z., Guo S., Lin Y. (2020). Effect of Qing’e Decoction on Leptin/Leptin Receptor and Bone Metabolism in Naturally Aging Rats. Evid.-Based Complement. Altern. Med..

[160] Xiong J., Cai X., Zhang Z., Li Q., Zhou Q., Wang Z. (2022). Elucidating the Estrogen-like Effects and Biocompatibility of the Herbal Components in the Qing’ E Formula. J. Ethnopharmacol..

[161] Zhang N.-D., Han T., Huang B.-K., Rahman K., Jiang Y.-P., Xu H.-T., Qin L.-P., Xin H.-L., Zhang Q.-Y., Li Y. (2016). Traditional Chinese Medicine Formulas for the Treatment of Osteoporosis: Implication for Antiosteoporotic Drug Discovery. J. Ethnopharmacol..

[162] Li B., Chen Z., Zhang Z., Liu H., Han D., Yang H., Zhang Z. (2024). Zuogui Pill Disrupt the Malignant Cycle in Breast Cancer Bone Metastasis through the Piezo1-Notch-1-GPX4 Pathway and Active Molecules Fishing. Phytomedicine.

